# The hemagglutinin-like proteins of basal vertebrate influenza-like viruses exhibit sialic-acid receptor binding disparity and their structural bases

**DOI:** 10.1371/journal.ppat.1013640

**Published:** 2025-11-26

**Authors:** Di Zhang, Kefang Liu, Yufeng Xie, Junqing Sun, Wei Zhang, Haixia Xiao, Yi Shi, William J. Liu, George F. Gao, Chuxia Deng, Feng Gao

**Affiliations:** 1 Faculty of Health Sciences, University of Macau, Macau SAR, China; 2 Beijing Life Science Academy, Beijing, China; 3 CAS Key Laboratory of Pathogen Microbiology and Immunology, Institute of Microbiology, Chinese Academy of Sciences (CAS), Beijing, China; 4 Department of Basic Medical Sciences, School of Medicine, Tsinghua University, Beijing, China; 5 Core Facility, Institute of Microbiology, Chinese Academy of Sciences, Beijing, China; 6 Laboratory of Protein Engineering and Vaccines, Tianjin Institute of Industrial Biotechnology, Chinese Academy of Sciences (CAS), Tianjin, China; 7 NHC Laboratory of Biosafety, National Institute for Viral Disease Control and Prevention, Chinese Center for Disease Control and Prevention (China CDC), Beijing, China; Icahn School of Medicine at Mount Sinai, UNITED STATES OF AMERICA

## Abstract

In 2018, two novel influenza-like virus genomes were first identified in basal vertebrates: the Asiatic toads (*Bufo gargarizans*) and spiny eels (*Mastacembelus aculeatus*). Their hemagglutinin (HA) proteins exhibit remarkably low amino acid sequences homology (23.0% and 42.8%, respectively) compared to influenza B virus (IBV), their closest canonical influenza virus relative. This study revealed that the Asiatic toad influenza-like virus HA (tHA) demonstrates dual receptor specificity, bound both α2–3 (avian-type) and α2–6 (human-type) sialic acid (SA) receptors, whereas the spiny eel influenza-like virus HA (eHA) lacks this capability. Biophysical characterization showed reduced thermal stability (lower T_m_ values) for both tHA and eHA compared to canonical influenza HA. Furthermore, we determined the cryo-EM structures of apo-tHA, tHA in complex with either α2–3 SA receptor or α2–6 SA receptor, as well as apo-eHA and eHA bound to GM2 complex. Our analysis revealed that tHA has a shorter length and looser HA trimer packing compared to canonical HA. These findings collectively indicate that influenza-like viruses in basal vertebrates have evolutionarily acquired dual SA receptor-binding capacity, a trait critical for cross-species transmission in influenza viruses. However, the observed thermolability of these HA proteins suggests that host physiological temperatures may impose a barrier to zoonotic spillover.

## Author summary

This study provides critical insights into the molecular mechanisms underlying the host receptor recognition of influenza-like viruses found in lower animals. We demonstrate that tHA binds to both α2–3 and α2–6 SA receptors, highlighting the potential for broader host range and cross-species transmission. In contrast, the eHA does not bind to these receptors. Structural analysis of tHA and eHA further reveals distinct conformational differences, including tHA’s shorter length and looser trimer packing. Additionally, our findings suggest that host temperature could play a key role in restricting virus transmission across species. These results emphasize the need for enhanced surveillance of influenza-like viruses in wildlife, as they may harbor zoonotic potential with the ability to interact with human and avian receptors.

## Introduction

Influenza virus poses a significant threat to global public health and economy [1–4]. Currently, there are four types of influenza viruses: A, B, C and D [5,6]. Influenza A viruses (IAVs) are the primary cause of seasonal flu and pandemics, whereas influenza B viruses (IBVs) mainly lead to seasonal flu outbreaks [7,8]. IAVs demonstrate a broad host range, spanning avian and mammalian species, exhibiting robust cross-species transmission potential, with water birds functioning as their primary natural reservoirs [9–13]. IBVs predominantly infect humans with low cross-species transmission capacity. But sporadic spillover events have been documented in seals [14]. In a recent report, IBVs were also detected in farmed bamboo rats in China [15]. Influenza C viruses (ICVs) primarily infect humans, and under natural conditions, there is also a potential for cross-species transmission between humans and pigs [16]. Influenza D viruses (IDVs) primarily infect cattle, occasionally transmitting to sheep or pigs [17,18].

The receptor binding activity of the HA protein is a key step for influenza virus infection and cross-species transmission [19–21]. Generally, avian influenza virus dominantly binds to α2–3 sialic acid (SA) receptor (avian receptor), while human influenza virus prefers to bind to α2–6 SA receptor (human receptor) [19,22,23]. However, bat-derived H17N10 and H18N11 subtype influenza-like viruses neither bind to α2–3 nor α2–6 SA receptor [24–26]. Previous study showed that the entry of bat-derived IAV-like virus is mediated by the major histocompatibility complex (MHC) class II molecule [27]. For IBVs, they can bind both α2–3 and α2–6 SA receptors [28].

In 2018, two novel influenza-like virus genomes were detected from the lungs of amphibian-Wuhan Asiatic toad (*Bufo gargarizans*) and the gill samples of ray finned fish-Wuhan spiny eel (*Mastacembelus aculeatus*), respectively [29]. The spiny eel influenza-like virus was identified with all eight genome segments, while Asiatic toad influenza-like virus, on the other hand, was identified with six intact genome segments, lacking M and NS segments [29]. HA protein phylogeny analysis showed that the spiny eel influenza-like virus and IBV form a sister-group. However, the HA genome of the Asiatic toad influenza-like virus cannot be classified into the evolutionary branch of either influenza A or B virus. The HA of the Asiatic toad influenza-like virus (tHA) and spiny eel influenza-like virus (eHA) share 23.0% and 42.8% amino acid sequences homology with IBV HA, respectively. It was revealed that eHA could bind to monosialic ganglioside 2 (GM2) and mediates fusogenicity under various pH conditions [30]. However, the structure of eHA and its complex with GM2 remain unknown, and it has not yet been evaluated whether tHA binds to α2–3 or α2–6 sialic acid receptors.

In 2020, a variety of influenza-like virus genome segments were further identified from various lower vertebrates through analysis of the available RNA sequencing database [31]. These findings suggest that the prevalence of influenza-like viruses in lower vertebrates may be a common phenomenon, with significant implications for the origin and evolution of influenza viruses. Furthermore, there is ongoing concern about whether these novel influenza-like viruses can infect humans or serve as new reservoirs for influenza viruses, potentially contributing gene segments to circulating influenza viruses in mammals and birds.

In this study, we elucidated the functional and structural characteristics of tHA and eHA. These findings offer a molecular understanding of HA proteins in influenza-like viruses from lower vertebrates, highlighting the potential risk of cross-species transmission of these two viruses.

## Results

### Biochemical and biophysical characterizations of soluble tHA and eHA

**Fig 1:**
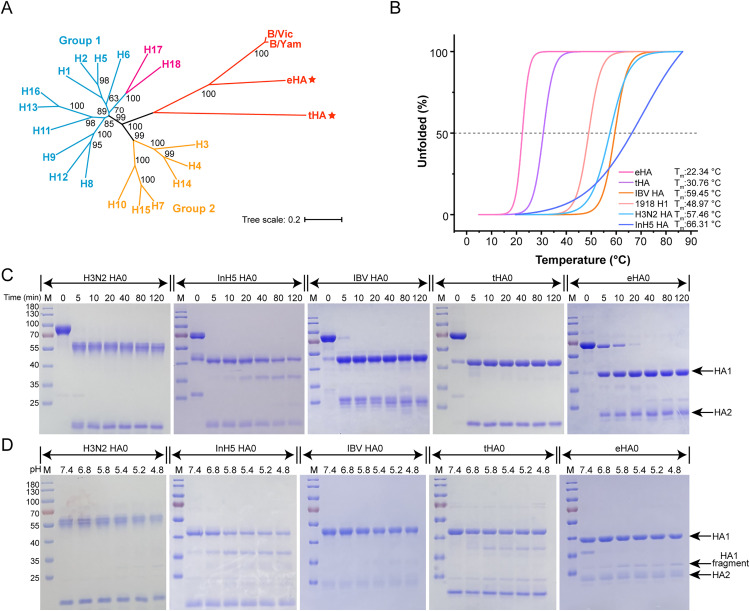
Biochemical and biophysical characterization of the soluble tHA and eHA proteins. **(A)** Phylogenetic tree of HAs from different influenza viruses. **(B)** Thermostability analyses of the tHA and eHA proteins with other characterized HA proteins (IBV HA (B/Austria/1359417/2021), 1918 H1, H3N2 HA and InH5 HA). **(C)** Trypsin susceptibility assays of soluble H3N2 HA, InH5 HA, IBV HA (B/Austria/1359417/2021), tHA and eHA. The tHA and eHA proteins can be digested into HA1 fragment and HA2 fragment at pH 8.0. As a control, the H3N2 HA, InH5 HA, IBV HA (B/Austria/1359417/2021) can be also cleaved into HA1 fragment and HA2 fragment at pH 8.0. **(D)** Low-pH digestion experiment of soluble H3N2 HA (A/Kansas/14/2017), InH5 HA, IBV HA (B/Austria/1359417/2021), tHA and eHA. The tHA and eHA proteins can be digested into different HA1 fragments and one HA2 fragment at low-pH. As a control, the low-pH-incubated H3N2 HA (A/Kansas/14/2017), InH5 HA and IBV HA (B/Austria/1359417/2021) proteins can be digested into different HA1 fragments and one HA2 fragment.

Phylogenetic analysis showed that both tHA and eHA are rooted in the same main branch of IBV HA. However, the genome of the Asiatic toad influenza-like virus cannot be classified into the evolutionary branch of either IAV or IBV (**Fig 1A and** **S1 Table**).

Firstly, the thermal stability of tHA and eHA were assessed through temperature-dependent circular dichroism (CD) spectroscopic experiment (**Fig 1B**).

The trypsin susceptibility assay was used to investigate the sensitivity of tHA and eHA to enzyme digestion. Our findings demonstrated that HA0 of both tHA and eHA were cleaved by trypsin into HA1 and HA2 (**Fig 1C**). As positive controls, HA0 of H3N2, InH5 and IBV were also cleaved into HA1 and HA2 by trypsin (**Fig 1C**). Further analysis of the cleavage site sequences revealed that tHA and eHA share a high degree of similarity with the cleavage site sequences of IAV and IBV (**S1 Fig**). Similar to H3N2 HA, InH5 HA and IBV HA, the cleaved tHA HA1 and eHA HA1 could be further cleaved into different fragments under low pH conditions ranging from 6.8 to 4.8 (**Fig 1D**). These results suggest that the tHA and eHA proteins demonstrate similar sensitivity to enzyme digestion as canonical HA proteins, despite their phylogenetical distance.

### Host tissue tropism and receptor-binding property of tHA and eHA

The apical surface of the human trachea primarily presents a variety of glycan receptors terminated by α2–6 SA linkages [32], while the apical surface of the duck intestinal tract predominantly showcases diverse glycan receptors terminated by α2–3 SA linkages [33]. To evaluate the potential risk of interspecies transmission of the Asiatic toad influenza-like virus and spiny eel influenza-like virus, we performed glycan microarray. The tHA protein exhibited binding avidity to glycans that were terminated with SA2,3 Gal. Unfortunately, the tHA protein did not display obvious avidity to glycans that were terminated with SA2,6 Gal (**S2 Fig**). We speculate that the observed lack of binding may be attributed to the low affinity of tHA for the α2–6 SA receptor. Through glycan array experiments, we have also confirmed that eHA cannot bind to either α2–3 or α2–6 SA receptors (**S2 Fig**), but it can bind to the GM2 receptor [30].

**Fig 2:**
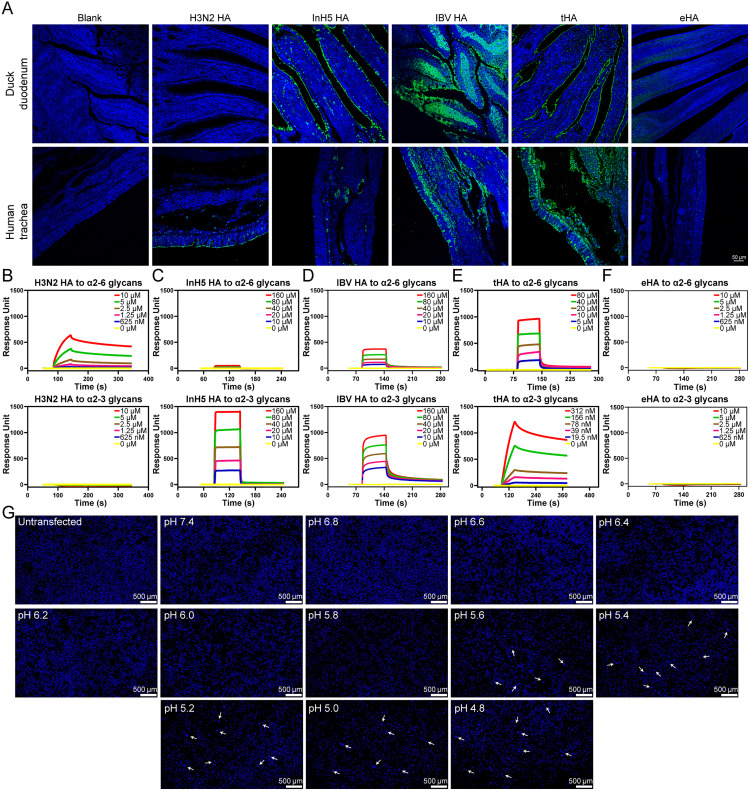
Host tissue tropism and receptor-binding properties of tHA and eHA. **(A)** Paraffinized tissue sections were stained with recombinant HAs derived from the baculovirus expression system. The specific staining of the recombinant protein was green. The nuclei were counterstained with DAPI (blue). **(B-F)** BIAcore diagram of HAs from different influenza viruses binding to α2-3 and α2-6 glycan receptors. The binding affinities of tHA (E) and eHA (F) to α2-3 and α2-6 glycans were measured using SPR assays. As controls, H3N2 HA (A/Kansas/14/2017) **(B)** InH5 HA (C) and IBV HA (B/Austria/1359417/2021) (D) were also assessed for their binding to α2-3 and α2-6 glycans. **(G)** A syncytia formation assay was applied to determine the membrane fusion of the tHA. BHK-21 cells transfected with pCAGGS plasmids expressing the tHA protein were treated with TPCK-treated trypsin, exposed to different pH conditions, and allowed to recover to determine for cell-to-cell fusion. All experiments were performed in triplicate, and one representative result is shown in Fig 2.

Then, we employed soluble recombinant HA proteins to stain paraffinized sections of human trachea and duck duodenum. Through immunofluorescence assay, we observed that tHA was able to stain both human trachea and duck duodenum, whereas eHA could stain neither of them (**Fig 2A**). As positive controls, the HA from A/Kansas/14/2017 (H3N2) was observed to exclusively stain the human trachea rather than the duck duodenum. In contrast, the InH5 HA selectively stained the duck duodenum but not the human trachea. And the IBV HA could stain not only the duck duodenum but also the human trachea.

The receptor-binding affinity of tHA to α2–3 and α2–6 SA receptors were tested by SPR assay. tHA binds to both α2–3 and α2–6 SA receptors, with affinities of 35.3 nM and 35.7 μM, respectively (**Fig 2E**). The 1000-fold lower binding affinity of tHA to the α2–6 SA receptor compared to the α2–3 SA receptor may explain why the tHA protein did not exhibit significant avidity to glycans terminated with SA2,6 Gal. However, we did not detect the binding of eHA to either α2–3 or α2–6 SA receptors (**Fig 2F**). As a positive control, HA from A/Kansas/14/2017 (H3N2) showed preferential binding to the α2–6 SA receptor (with an affinity of 4.68 μM) but lacks detectable binding to the α2–3 SA receptor (**Fig 2B**). InH5 HA bound to the α2–3 SA receptor with an affinity of 68.3 μM, but its binding to the α2–6 SA receptor was undetectable. (*K*_D_ > 1 mM, beyond the SPR measurement range) (**Fig 2C**) [34]. IBV HA binds to both α2–3 SA receptor and α2–6 SA receptor, with affinities of 29.8 μM and 85.5 μM, respectively (**Fig 2D**).

To assess the fusogenic potential of tHA, we conducted a syncytia formation assay under various pH conditions. Syncytia were observed across a range of pH values tested, from pH 4.8 to 5.6 (**Fig 2G**). No syncytia formation was observed at the neutral pH of 7.4 or in untransfected cells.

Additionally, we evaluated the receptor binding activity of tHA and eHA proteins through hemagglutination assay with chicken and guinea pig red blood cells (RBCs). tHA displayed hemagglutination activities similar to those of the H3N2, InH5 and IBV HAs when incubated with 1% chicken or 2% guinea pig RBCs at an initial concentration of 10 μg (**S3 Fig**). Whereas, eHA did not exhibit hemagglutination activity (**S3 Fig**), consistent with no SA-binding activity.

### Overall architectures of both tHA and eHA

**Fig 3:**
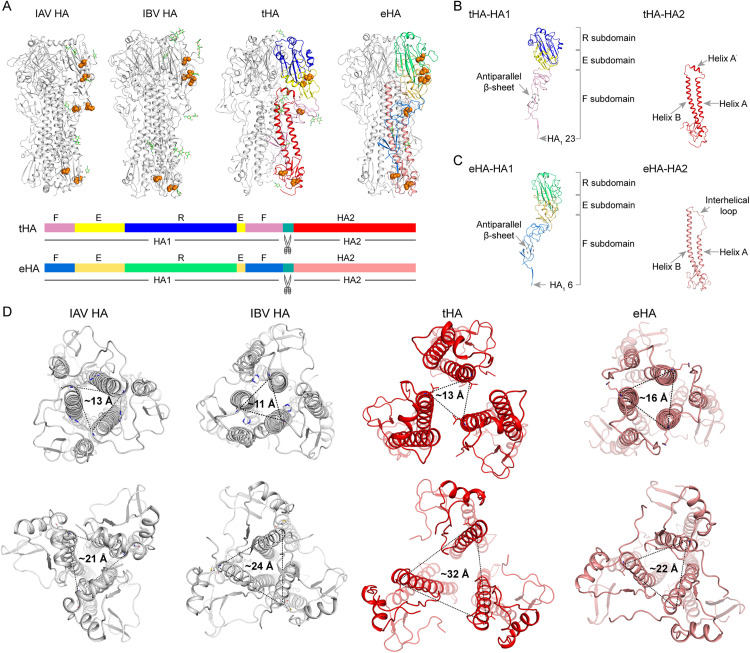
Overall structure of the tHA and eHA proteins. **(A)** Comparison of the trimers among tHA, eHA, IAV and IBV HA. For IAV HA A/Singapore/H2011.447/2011 PDB code:4WE8) and IBV HA (B/Brisbane/60/2008 PDB code: 4FQM), the trimers are colored gray. One monomer of tHA and eHA are marked with different colors according to the domain. N-linked glycans are highlighted in sticks (green) and disulfide bonds are displayed in orange. **(B)** The HA1 domain component of the tHA: the receptor-binding subdomain (R subdomain, blue), the vestigial esterase subdomain (E subdomain, yellow), the fusion subdomain (F subdomain, pink). The HA2 domain from tHA shows the helix A, helix B and helix A’ (red). **(C)** The HA1 domain component of the eHA: R subdomain (green), E subdomain (lemon yellow), F subdomain (marine). The HA2 domain from eHA shows the helix A, helix B and interhelical loop (pink). **(D)** Comparison of the distance between the helices formed by the longer α-helix B in HA2. IAV HA2 and IBV HA2 are colored gray, tHA HA2 is colored red, and eHA HA2 is colored pink.

As described above, tHA and eHA share low sequence homology with canonical HAs, yet retain certain characteristic features of the canonical HA proteins (**S1 Table**). To elucidate the molecular mechanism, the cryo-EM structures of the extracellular domain of apo-tHA and apo-eHA were determined at resolutions of 2.65 Å and 2.95 Å, respectively (**S4** **and** **S5 Figs** **and** **S6 Table**). The tHA and eHA resembles the typical homotrimeric conformation (**Fig 3A**). As the description of the IAV HA structure [35], the HA1 of tHA and eHA are divided into a receptor binding region subdomain (R subdomain), a vestigial esterase subdomain (E subdomain), a fusion subdomain (F subdomain) and HA2 is a membrane anchor subdomain (**Fig 3B and 3C**).

Compared to the HAs of IAV and IBV, the overall structures of tHA and eHA exhibit significant differences (**S2 Table**). The root-mean-square deviations (RMSDs) of tHA to IAV HA and IBV HA are 29.621 Å and 29.890 Å for all Cα atoms, respectively, while the RMSDs of eHA are 6.332 Å and 3.256 Å for all Cα atoms, respectively (**S6A-D Fig**). Although the sequence identity of tHA and eHA subdomains to their counterparts in IAV or IBV HA is notably low, their tertiary structures exhibit high similarity. Nonetheless, differences in secondary structure are observed in each subdomain. The RMSDs of tHA subdomains to their counterparts in IAV or IBV range from 2.974 Å to 4.712 Å, while the RMSDs of the eHA subdomain to their counterparts in IAV or IBV range from 0.795 Å to 4.895 Å (**S6A-D Fig**).

In canonical HA, three short helices (α-helix A) attach to α-helix B in an antiparallel manner, twisting around it. In its native state, these helices are linked by an interhelical loop, transitioning into a helical structure during HA’s conformational change in membrane fusion. In eHA, α-helix A and α-helix B are connected by an interhelical loop, similar to canonical HA. However, in tHA, the interhelical loop between α-helix A and α-helix B is replaced by an α-helix, termed helix A’. The lengths of α-helix A and α-helix B are comparable (**S6A** and **S6B Fig**).

Traditionally, the R subdomain in canonical HAs comprises an antiparallel β-sheet, typically forming an 8-strand Swiss-roll structure (**S7C** and **S7D Fig**). In contrast, the R subdomain conformation in both tHA and eHA features a 7-strand antiparallel β-sheet structure (**S7A** and **S7B Fig**).

**Fig 4:**
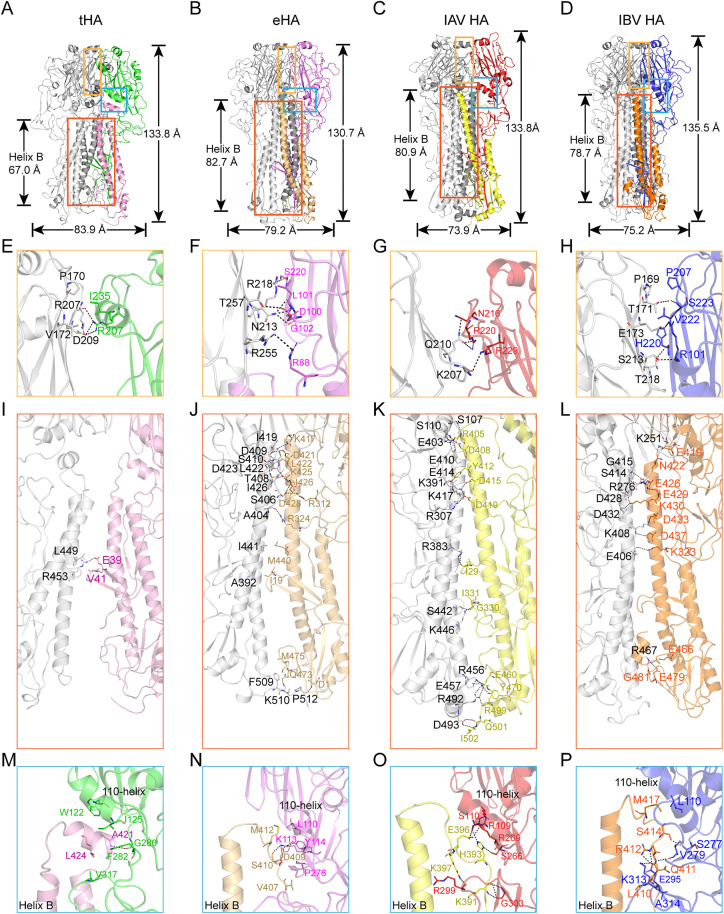
Overall structures of the tHA, eHA, IAV (A/Singapore/H2011.447/2011 PDB code:4WE8) and IBV (B/Brisbane/60/2008 PDB code: 4FQM). **(A-D)** Overall structures of the tHA, eHA, IAV HA and IBV HA. **(E-H)** The HAs of tHA, eHA, IAV and IBV interactions between HA1 residues with the adjacent HA monomer molecule. The hydrogen bond is shown in a dash line. **(I-L)** The HAs of tHA, eHA, IAV and IBV interactions between HA2 residues with the adjacent HA monomer molecule. The hydrogen bond is shown in a dash line. **(M-P)** The HAs of tHA, eHA, IAV and IBV interactions between residues in the interhelical loop (tHA: helix A’), 110-helix of E subdomain and the F subdomain. The hydrogen bond is shown in a dash line.

Overall, the height of tHA is 133.8 Å which is comparable to IAV HA (133.8 Å) and slightly shorter than IBV HA (135.5 Å). The width of tHA (83.9 Å) is obviously wider than both IAV HA (73.9 Å) and IBV HA (75.2 Å) (**Fig 4A**, **4C and 4D**). The length of tHA helix B (67.0 Å) is significantly shorter than those of IAV (length: 80.9 Å) and IBV (length: 78.7 Å) (**Fig 4A**, **4C and 4D**). The overall packing of tHA is looser than that of IAV and IBV. The height of eHA is 130.7 Å which is slightly shorter than both IAV HA (133.8 Å) and IBV HA (135.5 Å). The width of eHA (79.2 Å) is obviously wider than both IAV HA (73.9 Å) and IBV HA (75.2 Å) (**Fig 4B**, **4C and 4D**). The length of eHA helix B (82.7 Å) is significantly longer than those of IAV (length: 80.9 Å) and IBV (length: 78.7 Å) (**Fig 4B**, **4C and 4D**). The overall packing of eHA is slightly looser than that of IAV and IBV, but more compact than that of tHA.

Then, we analyzed the interactions between the different monomers of the tHA and eHA trimer. For IAV HA and IBV HA, the major forces stabilizing hemagglutinin trimer interactions arise from the HA2 region. In HA2, the α-helix B sub-domain of each HA monomer assembles to form a triple coiled coil that close together at its amino terminus, with an interhelical distance of approximately 10 Å. The three helices twist around each other and spread out in the vicinity of viral envelope (about 22 Å between the helices), with polar and charged residues interacting around the 3-fold helices (**Figs 3D**, **4K and 4L**) [36]. In addition, both IAV and IBV also exhibit a lot of hydrophobic interactions at this position (**S3** **and** **S4 Tables**).

Different from IAV and IBV, the HA1 subunit of tHA exhibits significantly more intra-subunit interactions than HA2. In the HA1 of tHA, residues R207 and D209 form salt bridge interactions with residue R207 of adjacent HA1 monomer, while hydrophobic interactions occur between residue I235 of HA1 and residues P170 and V172 of the neighboring HA1 monomer (**Fig 4E**). Conversely, interactions at the tHA HA2 trimer interface are notably fewer. Only a solitary hydrogen bond forms between residue R453 of HA2 and residue E39 of the adjacent monomer F subdomain, along with a hydrophobic interaction between residue L449 of HA2 and residue V41 of the adjacent monomer F subdomain (**Fig 4I**). Consequently, the structural integrity of the tHA trimer is likely maintained predominantly by the globular head. Additionally, interactions within the HA2 region of tHA are weak, resulting in a distance of 32 Å between the helices near the virus envelope (**Fig 3D**). For the eHA, the HA2 subunit of eHA exhibits significantly more intra-subunit interactions than HA1 (**Fig 4F and 4J**). Consequently, the structural integrity of the eHA trimer is likely maintained predominantly by the HA2 subunit. The interactions between the different monomers of the eHA trimer are weaker than those of IAV and IBV, but stronger than those of tHA.

We also meticulously investigated the interactions between HA1 and HA2 in the tHA monomer and eHA monomer, particularly focusing on residues in the helix A’ (eHA: interhelical loop), the 110-helix of the E subdomain, and the F subdomain, in comparison to IAV and IBV HA monomers. In IAV, a hydrogen bond is established between residue S110 of the 110-helix and residue H393 of the interhelical loop. Additionally, residue E396 of the interhelical loop forms salt bridges with residues R109 and R269 of the E subdomain (**Fig 4O**). In IBV, residues S414 and Q411 of the interhelical loop form hydrogen bonds with residues S277 and K313 of HA1, respectively. Furthermore, residues R412 of the interhelical loop and E295 of HA1 form a salt bridge (**Fig 4P**). Notably, hydrophobic interactions occur between residue M417 and residue L110, as well as between residue L410 and residue A314 (**Fig 4P**). These interactions collectively contribute to the conformational stability of HA1 and HA2. In tHA, only residue A421 of the helix A’ and residue G280 of HA1 form a hydrogen bond. However, tHA exhibits numerous hydrophobic interactions at this position, involving residues (W122 and I125) from the 110-helix, residues (F282 and V317) from the F subdomain, and residues (A421 and L424) from the helix A’ (**Fig 4M**). In eHA, residues M412 and V407 of the interhelical loop form hydrophobic interactions with residues L110 and P276 of HA1, respectively (**Fig 4N**). Moreover, hydrogen bonds are formed between residue D409 and residue S410, as well as between residue Y114 and residue K113 (**Fig 4N**).

### Structural basis for tHA binding to the canonical SA receptors

To explore the molecular mechanism of receptor binding of tHA, the two sialo-pentasaccharides, the α2–3 glycans (3’SLNLN: NeuAcα2–3Galβ1–4GlcNAcβ1–3Galβ1–4Glc) and the α2–6 glycans (6’SLNLN: NeuAcα2–6Galβ1–4GlcNAcβ1–3Galβ1–4Glc), were incubated with tHA protein. The complex structures were determined at resolutions of 2.37 Å and 2.78 Å, respectively (**S8** **and** **S9 Figs** **and** **S6 Table**).

**Fig 5:**
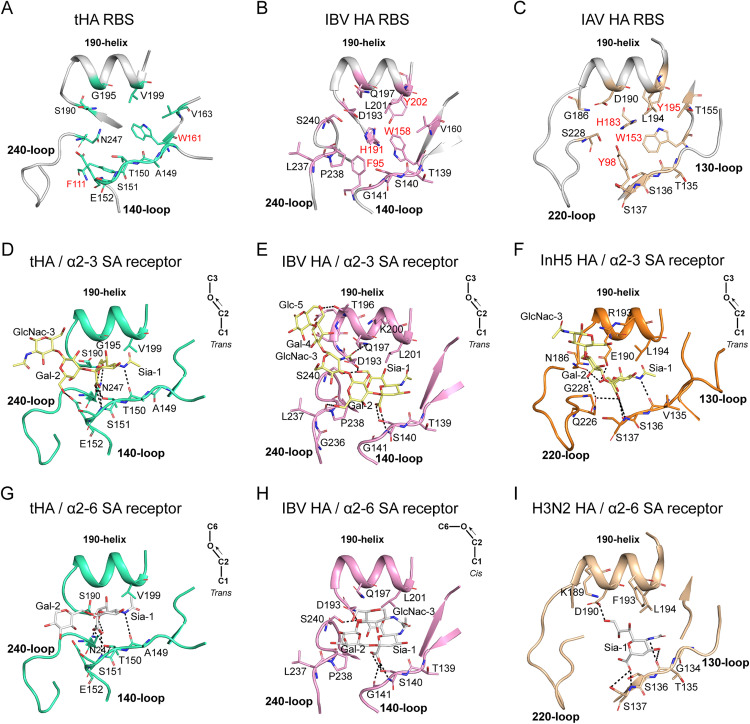
Structural comparative analyses of the interactions of tHA, IBV HA (B/Hong Kong/8/1973 PDB code: 2RFT) and IAV HA (A/Singapore/H2011.447/2011 PDB code: 4WEA) with either avian or human receptor analogs. **(A-C)** Schematic representation of the tHA, IBV and IAV HA RBS with sticks representing key residues for receptor binding. The conserved residues that form the base of the RBS are marked in red. **(D-F)** The three secondary structural elements of the binding site (i.e., the 140-loop, 190-helix, and 240-loop) are labeled in ribbon representation, together with selected residues in stick representation. The hydrogen bonds are shown as dashed lines. The tHA is colored in greencyan, the IBV HA (PDB code: 2RFT) is colored in pink and the InH5 HA (PDB code: 4K63) is colored in tv-oranges. The avian receptor analogs are colored paleyellow. (D to F) tHA (D), IBV HA (E) and InH5 HA (F) with the avian receptor analogs bound. **(G-I)** The tHA is colored in greencyan, the IBV HA (PDB code: 2RFU) is colored in pink and the H3N2 HA (PDB code: 4WEA) is colored in wheat. The human receptor analogs are colored white. (G to I) tHA (G), IBV HA (H) and H3N2 HA (I) with the human receptor analogs bound.

Similar to the canonical HA receptor binding site (RBS), the RBS of tHA is located at top of the globular domain of tHA, consisting of three secondary structural elements the 140-loop (IBV: 140-loop, IAV: 130-loop), the 240-loop (IBV: 240-loop, IAV: 220-loop) and 190-helix (IBV: 190-helix, IAV: 190-helix) (**Fig 5A**, **5B and 5C**). In IBV HA, four amino acids form the base of the groove: F95, W158, H191 and Y202 (**Fig 5B**). In IAV, the base of the groove is formed by four highly conserved amino acids (Y98, W153, H183 and Y195) (**Fig 5C**). However, only two amino acids, F111 and W161, form the base of the groove at the RBS of tHA (**Fig 5A**).

In the complex structure of tHA with the α2–3 SA receptor, the electron density of the Sia-1, Gal-2 and GlcNAC-3 moieties is clearly discernible (**S10A Fig**). The avian receptor analog binds tHA in a *trans* conformation (**Fig 5D**). Compared to the IBV and IAV HA receptor complex, the α2–3 SA receptor bound to tHA is distinct [28,34,37]. In the structure of tHA in complex with the α2–3 SA receptor, residues A149, T150 and S151 of the 140-loop form hydrogen bonds with the Sia-1 (**Fig 5D**). Conversely, in the IBV/α2–3 SA receptor structure, two hydrogen bonds are formed between the Sia-1 and the 140-loop residues (S140 and G141), while residue T139 of the 140-loop interacts via van der Waals forces with the Sia-1 (**Fig 5E**). In the IAV InH5 HA/α2–3 SA receptor structure, four hydrogen bonds are formed between the Sia-1 and the 130-loop residues (V135, S136 and S137) (**Fig 5F**). Notably, residue E152 in the 140-loop of the tHA forms a hydrogen bond with the Gal-2, which is not present in IBV and IAV (**Fig 5D**), which explains why tHA shows significantly higher affinity for SA 2–3 Gal compared to avian virus HAs. For the 240-loop, residue N247 forms two hydrogen bonds with the Sia-1 in tHA (**Fig 5D**). In the IBV/α2–3 SA receptor structure, S240 forms a hydrogen bond with the Sia-1, and L237 forms a hydrogen bond with the Gal-2 (**Fig 5E**). In the InH5 HA/α2–3 SA receptor structure, residue Q226 of the 220-loop forms two hydrogen bonds with Sia-1 and Gal-2 (**Fig 5F**). For the 190-helix, residues S190, G195 and V199 of tHA only form van der Waals force with the Sia-1, and no hydrogen bond is formed (**Fig 5D**). In contrast, the amino acid residues of the 190-helix from IBV and IAV HAs establish hydrogen bonds with the α2–3 SA receptor (**Fig 5E** and **5F**).

In the complex structure of tHA with the α2–6 SA receptor, only the Sia-1 and Gal-2 moieties exhibit clear electron density (**S10B Fig**). Differing from the *cis* conformation of the α2–6 SA receptor in complex with IBV HA, the α2–6 SA receptor binds to tHA in a *trans* conformation (**Fig 5G**-5I). Within the structure of tHA in complex with the α2–6 SA receptor, residues A149, T150 and S151 from the 140-loop, along with residue N247 from the 240-loop form hydrogen bonds with the Sia-1, whereas residue E152 from the 140-loop engages in van der Waals interactions with the Gal-2 (**Fig 5G**). This disparity likely contributes to the higher affinity of tHA for the α2–3 SA receptor compared to the α2–6 SA receptor, attributed to the absence of a hydrogen bond formed between E152 and Gal-2. Concerning the 190-helix, residues S190 and V199 only form van der Waals interactions with the Sia-1 (**S5 Table**). Conversely, in the IBV/α2–6 SA receptor and IAV/α2–6 SA receptor complex structures, residues of 140-loop (IAV: 130-loop) form hydrogen bonds with the Sia-1, and D193 (IAV: D190) of the 190-helix also forms hydrogen bond with the Sia-1 (**Fig 5H** and **5I**). We observed that tHA’s binding capability to the receptors primarily relies on the amino acids from the 140-loop, with E152 playing a pivotal role in its strong affinity for the avian receptor.

### Complex structure of eHA bound to GM2

**Fig 6:**
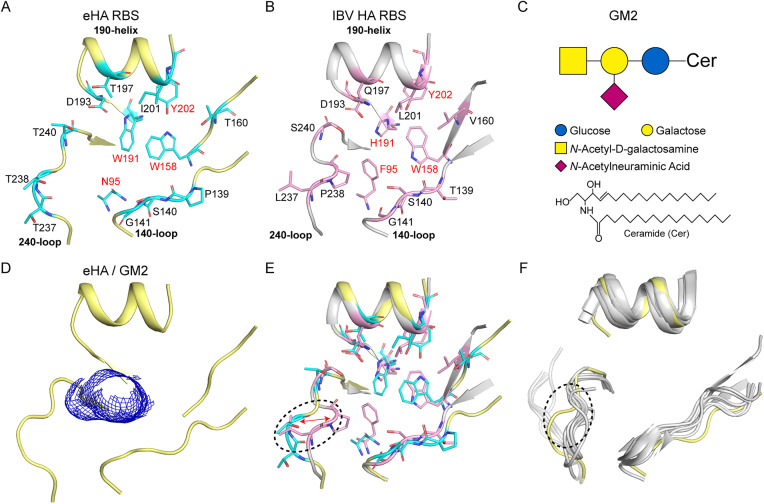
Comparative structural analyses of the interactions of eHA and IBV HA (B/Hong Kong/8/1973 PDB code: 2RFT). **(A-B)** Schematic representation of the eHA and IBV HA RBS with sticks representing key residues for receptor binding. The conserved residues that form the base of the RBS are marked in red. **(C)** The schematic of GM2. **(D)** Cryo-EM density maps of the GM2 in the eHA-GM2 complex **(E)** Comparison of eHA and IBV RBS. **(F)** Comparison of eHA, IAV (gray: H1, H2, H3, H5, H9, H14, H16) and IBV RBS.

While the amino acid sequence of eHA is only 42.8% identity to IBV HA, the overall structure of eHA is very similar to IBV HA, with an RMSD of 3 Å (**S1** **and** **S2 Tables**). The RBS of eHA is located at top of the globular domain of eHA, consisting of three secondary structural elements: the 140-loop (IBV: 140-loop), the 240-loop (IBV: 240-loop) and the 190-helix (IBV: 190-helix) (**Fig 6A**). In IBV HA, four amino acids form the base of the groove: F95, W158, H191 and Y202 (**Fig 6B**). In eHA, the base of the groove is formed by N95, W158, W191 and Y202 (**Fig 6A**). Previous studies have demonstrated that eHA could bind to GM2. We observed cryo-EM density maps of GM2 in the eHA-GM2 complex, where significant unmodeled electron density was detected at the RBS (**Figs 6C**, **6D, and** **S11**).

Although there is a noticeable cavity at the RBS of eHA, this region exhibits a slight negative charge, which repels the negative charge of SA (**S12 Fig**). Additionally, compared to IBV HA, the P238 in the 240-loop corresponds to T238 in eHA, and this change causes an outward shift in the 240-loop (**Fig 6E**). Furthermore, we compared the RBS of all canonical IAV and IBV strains with that of eHA and found that only the 240-loop of eHA RBS is outward shift (**Fig 6F**). These factors collectively contribute to eHA’s inability to bind to canonical SA receptors.

## Discussion

Despite tHA and eHA share 23.0% and 42.8% amino acid sequences homology with IBV HA, respectively, both tHA and eHA retain the canonical features of HA but exhibit significant structural differences. We observed the cleavage of tHA and eHA precursor protein HA0 into HA1 and HA2 by trypsin. Furthermore, low-pH digestion experiments revealed that cleaved tHA and eHA can undergo activation under acidic conditions, leading to membrane fusion-conducive conformational changes. These findings indicate that tHA and eHA exhibit biochemical and biophysical properties akin to canonical HA proteins.

Notably, in a recent study, it was found that the eHA can facilitate fusogenicity across a range of pH conditions [30]. The eHA specifically binds to a unique GM2 gangliosidic receptor, which has been identified as an interacting partner of some reoviruses and rotaviruses [30]. Herein, we observed the density map obtained after incubating GM2 with eHA, although the map quality is suboptimal. The unclear eHA structural interpretation is likely due to the poor solubility of GM2, which hindered the determination of its precise three-dimensional structure. Moving forward, we will focus on acquiring higher-resolution datasets to accurately resolve the binding mode.

Additionally, SPR and immunofluorescence assays confirmed tHA’s ability to bind to both avian and human receptors. However, tHA displays lower thermostability and is prone to conformational changes, with 50% unfolding occurring at 30.76 °C. Asiatic toads’ body temperatures can drop to match ambient temperatures. The optimum growth temperature of Asiatic toad is 20–32 °C [38], facilitating the thriving of the Asiatic toad influenza-like virus in such conditions. In contrast, the typical resting value for deep tissue in the chest region of the human body temperature is 36–38 °C [39]. The body temperature of adult poultry is usually in the range of 41–42 °C [40]. The body temperature of human and poultry exceed 30.76 °C. Consequently, the Asiatic toad influenza-like virus may struggle to survive in these hosts due to temperature restriction. These data suggest that although tHA can bind to both avian and human receptors, further evolution is necessary to adapt to the higher body temperatures of humans and birds.

Our group recently reported that both Asiatic toad influenza-like virus neuraminidase (tNA) and spiny eel influenza-like virus NA (eNA) exhibit canonical sialidase activity [41,42]. However, tNA shows strong resistance to NA inhibitors, while eNA has no resistance to NA inhibitors [41]. Herein, we discovered that tHA also possesses similar functions to those of canonical influenza viruses. It is crucial to assess the infectivity of the authentic virus; however, the authentic virus of Asiatic toad influenza has not yet been isolated.

In conclusion, our data indicate that tHA can bind to both human and avian receptors, and we elucidated the structural basis of their interactions. Additionally, we studied the structure and function of eHA. We also found that host physiological temperature is an important factor limiting the interspecies transmission of influenza-like viruses.

## Methods

### Ethics statement

The paraffinized human tracheal tissue was provided by the China-Japan Friendship Hospital and approved by Clinical Research Ethics Committee of China-Japan Friendship Hospital. The ethics certificate number is 2022-KY-148. The paraffinized duck small intestine tissue sections were purchased from Auragene Bioscience Corporation.

### Cloning, expression and purification

As our previously reported the genes encoding the ectodomain of HA from Asiatic toad influenza-like virus (GenBank accession No. MG600048.1) and the spiny eel influenza-like virus (GenBank accession No. AVM87624.1) were cloned into the baculovirus transfer vector pFastBac1 (Invitrogen), respectively [43–45]. Transfection and virus amplification procedures followed the Bac-to-Bac baculovirus expression system manual (Invitrogen). HAs of A/Kansas/14/2017 (H3N2 HA), A/South Carolina/1/1918 (1918 H1), A/Indonesia/5/2005 (InH5 HA) and B/Austria/1359417/2021 (IBV HA) were prepared as previously described methods [34,46].

HA proteins were produced by infecting suspension cultures of High Five cells for 2 days. The proteins were purified using a His-Trap HP column (Cytiva), then purified by ion-exchange chromatography using a Resource Q 6 mL column (Cytiva). For structural and functional assays, the proteins were further purified by gel filtration chromatography using a Superdex-200 16/60 GL column (Cytiva) with a running buffer for 20 mM Tris-HCl and 50 mM NaCl (pH 8.0).

### Cryo-EM sample preparation and data collection

A total 5 μL purified tHA protein at the concentration of 8 mg/mL was applied for cryo-EM grid preparation. An aliquot of 4 μL protein sample of tHA was applied onto a glow-discharged 300 mesh grid (Quantifoil Au R1.2/1.3) after dilution. Grids were supported with a thin layer of GO (Graphene Oxide), blotted with filter paper for 2 s and plunge-frozen in liquid ethane using a Thermo Fisher Vitrobot Mark IV. Cryo-EM micrographs were collected on a 300kV Thermo Fisher Krios G4 electron microscope equipped with a Falcon 4 direct detection camera. The micrographs were collected at a calibrated magnification of x96,000, yielding a pixel size of 0.86Å at counted mode. In total, 4,818micrographs were collected at an accumulated electron dose of 49e^-^Å^-2^ s^-1^ on each micrograph that was fractionated into a stack of 32 frames with a defocus range of -1.0 μm to −2.0 μm.

For the tHA/α2–3 SA receptor complex, a 3 μL protein sample at approximately 2 mg/mL containing 10 mM glycan was applied to glow-discharged carbon holey grids (Quantifoils Au R1.2/1.3). The blotted grids (4 s, 100% humidity and 4 °C) were rapidly frozen in liquid ethane (Vitrobot Mark IV, Thermo Fisher Scientific). The movie stacks were collected on a 300 kV Titan Krios transmission electron microscope equipped with a Gatan K3 detector and GIF Quantum energy filter in super-resolution counting mode (magnification of 105,000 × , physical pixel size of 0.85 Å) using E Pluribus Unum (EPU). Each movie was dose-fractionated into 32 frames with a total dose of 60 e^-^/Å^2^ (defocus range of -1.0 to -2.0 µm). 7,249 movie stacks were collected.

The tHA/α2–6 SA receptor complex dataset was processed similarly. 300-mesh amorphous nickel-titanium alloy (ANTA) foil (1.2/1.3) Au grids (Zhenjiang Lehua Electronic Technology, China) and graphene oxide (GO) grids (GO on Quantifoils R1.2/1.3 300 mesh copper grids, R1.2/1.3) were selected for sample preparation. 5,310 movie stacks on the GO grids were collected while 1,765 movie stacks on the ANTA foils.

The cryo-sample of eHA protein dataset was processed similarly, a 4 μL protein sample at approximately 0.5 mg/mL was applied to GO grids (R1.2/1.3 300 mesh). The movie stacks were collected on a 300 kV Titan Krios transmission electron microscope equipped with a Gatan K3 detector and GIF Quantum energy filter in super-resolution counting mode (magnification of 105,000 × , physical pixel size of 0.69 Å) using EPU. Each movie was dose-fractionated into 32 frames with a total dose of 60 e^-^/Å^2^ (defocus range of -1.0 to -2.0 µm).

For the eHA/GM2 receptor complex, a 4 μL of eHA/GM2 complex sample at 0.41 mg/mL was applied to GO grids (R1.2/1.3 300 mesh). The above grids were then blotted using different conditions, i.e., blot time 2 s and blot force -2, at a temperature of 4 °C and a humidity level of >99% and plunge frozen into liquid ethane. The movie stacks were collected on a 300 kV Titan Krios transmission electron microscope equipped with a Gatan K3 detector and GIF Quantum energy filter. Movies were collected at 85,000 × magnification with a calibrated pixel size of 0.89 Å over a defocus range of -1.0 μm to -2.0 μm in super resolution counting mode with a total dose of 50 e^-^/Å^2^ using EPU automated acquisition software for eHA/GM2 complex sample.

### Image processing and 3D reconstruction

The detailed data processing workflow is summarized in S4, S5, S8, S9 and S11 Figs. Beam-induced motion correction was performed on the stack of frames using MotionCorr2 [47]. The contrast transfer function (CTF) parameters were determined by CTFFIND4 [48]. A total 4,818 good micrographs were selected for further data processing using cryoSPARC [49].

For the tHA protein, particles were auto-picked by the Auto-picking program in cryoSPARC, followed by 2 rounds of reference-free 2D classifications. Next, 1,470,313 particles were selected from good 2D classes and were subjected to a round of 3D classification using a reconstruction of the tHA protein as a starting model. A converged 3D class with a feature contains the trimer structure of tHA protein were selected for a final round of 3D refinement. 1,164,981 particles from the selected 3D class shows the highest resolution feature of tHA protein were selected for 3D refinement, with C3 symmetry applied, yielding a final reconstruction at a global resolution of 2.65 Å based on the gold-standard Fourier shell correlation criterion at Fourier shell correlation (FSC) = 0.143. The local resolution was then calculated on the final density map.

For the tHA/α2–3 SA receptor complex, 7,249 movies stacks were collected. The movies were motion corrected using MotionCor2. The contrast transfer function (CTF) parameters were estimated using patch CTF estimation. Blob picking in a subset of 3,631 yielded 8,896,804 particles, and 2D classification separated out 15,291 clean particles for Topaz training. Topaz extraction yielded a total of 646,713 particles which were extracted and subjected to 2D classification. After 2D classification, 175,064 clean particles were selected to perform initial reconstruction and heterogeneous refinement. The particles from the best converged 3D class were select to repeat Topaz training and extraction, which augment the dataset to 700,575 particles. Iterative 2D classification and two rounds of heterogeneous with C3 symmetry seperated out 119,048 particles. Homogeneous refinement with C3 symmetry yielded a final reconstruction at a global resolution of 2.37 Å determined by the FSC = 0.143 criterion.

The tHA/α2–6 SA receptor complex dataset was processed similarly. The final reconstruction yielded a volume map at a global resolution of 2.78 Å with C1 symmetry determined by the FSC = 0.143 criterion.

For the eHA protein dataset was processed similarly. The final density map at 2.95 Å resolution with C3 symmetry determined by the FSC = 0.143 criterion.

For the eHA/GM2 receptor complex dataset was processed similarly. The final reconstruction yielded a volume map at a global resolution of 2.75 Å with C1 symmetry determined by the FSC = 0.143 criterion. To improve the local resolution of the GM2 domain, we generated a mask around the GM2 domain and performed local refinement.

### Model building and refinement

For the initial model building of the tHA, the wild-type InH5 HA (PDB code: 4K63) was used as the stalk domain starting model of the tHA and fitted into the corresponding overall cryo-EM maps using UCSF Chimera v.1.15 [50]. Our model of the globular domain of tHA was manually built according to our cryo-EM density map using the Phenix autobuild and COOT v.0.9.3 [51]. The complex structures were subsequently determined using the refined tHA as input models. The eHA (PDB code: 4SJ9) was rigidly fitted into the cryo-EM map using UCSF Chimera v.1.15. Mutation and manual adjustment were carried out with Coot v.0.9.3. The eHA/GM2 receptor complex structure was subsequently determined using the refined eHA as input models. Most residue side chains were clearly visible in the map. Each residue was manually checked with the chemical properties taken into consideration during model building. Several rounds of the real-space refinement in Phenix [52] and manually building in COOT were performed until the final reliable models were obtained. Molprobity [53] was used to validate geometry and check structure quality. Statistics associated with data collection, 3D reconstruction and model building were summarized in S6 Table. Figures were generated using PyMOL (http://www.pymol.org/).

### SPR analysis

The affinity and binding kinetics of HAs to receptor analogs were measured at 25 °C on a BIAcore 3000 machine (GE Healthcare) with streptavidin chips (SA chips, GE Healthcare). For all measurements, PBST (phosphate buffered saline (PBS) buffer with 0.005% Tween 20, pH 7.4) was used as the running buffer. All of the tested proteins were stored in the PBST buffer. Two biotinylated receptor analogs, the α2–3 glycans (3′SLNLN: NeuAcα2–3Galβ1–4GlcNAcβ1–3Galβ1–4GlcNAcβ1-SpNH-LC-LC-Biotin) and the α2–6 glycans (6′SLNLN: NeuAcα2–6Galβ1–4GlcNAcβ1–3Galβ1–4GlcNAcβ1-SpNH-LC-LC-Biotin) were kindly provided by Consortium for Functional Glycomics (Department of Molecular Biology, Scripps Research Institute). Approximately 400 response units of biotinylated glycans were immobilized on the chip and a blank channel was used as the negative control. H3N2 HA was serially diluted to concentrations ranging from 0.625 μM to 10 μM and were then flowed over the α2–3 glycans and α2–6 glycans channels, respectively. The concentrations ranging from 10 μM to 160 μM of InH5 HA were flowed over the α2–3 glycans and α2–6 glycans channels, respectively. The concentrations ranging from 10 μM to 160 μM of IBV HA were flowed over the α2–3 glycans and α2–6 glycans channels, respectively. The concentrations ranging from 5 μM to 80 μM of tHA were flowed over the α2–6 glycans channel. The concentrations ranging from 19.5 nM to 312 nM of tHA were flowed over the α2–3 glycans channel. The concentrations ranging from 625 nM to 10 μM of eHA were flowed over the α2–3 glycans and α2–6 glycans channels, respectively. The sensor surface was regenerated with 10 mM NaOH at the end of each cycle. Sensograms were locally fitted with BIAcore3000 analysis software (BIAevaluation Version 4.1) using a 1:1 Langmuir binding model. The affinity values of the InH5 HA to α2–3 glycans, IBV HA to α2–3 glycans, IBV HA to α2–6 glycans and tHA to α2–6 glycans were calculated with a steady state affinity model due to the fast *K*_*on*_ and *K*_*off*_. The affinity values of the H3N2 HA to α2–6 glycans and tHA to α2–3 glycans were calculated with a simultaneous kinetic *K*_a_ (association rate)/ *K*_d_ (dissociation rate) model.

### Binding of HAs to human and duck tissue sections

The immunofluorescence assay was based on those reported by Chandrasekaran *et al*. and Tharakaraman *et al* [54,55]. Briefly, paraffinized human tracheal and duck small intestine tissue sections were deparaffinized, rehydrated and incubated with 4% bovine serum albumin (BSA) in PBS for 30 min at room temperature (RT) to prevent nonspecific binding. The HA protein, primary antibody (mouse anti-His-tag, EASYBIO) and secondary antibody (Alexa Fluor 488 goat anti-mouse IgG, Invitrogen) at a molar ratio of 4:2:1, respectively. The tissue sections were incubated with HA protein (100 μg/mL) at RT for 1 h. Then, the primary antibody was applied to the tissue sections and incubated at RT for 1 h. Subsequently, the secondary antibody was applied to the tissue sections and incubated at RT for 40 min. After each incubation step, the tissue sections were washed three times with PBS for 5 min each. Sections were counterstained with diamidino-2-phenylindole (DAPI) for nuclei at RT. After 30 min incubation, the sections were washed three times with PBS for 5 min each time. Then the tissue sections were mounted and then examined by a confocal laser scanning microscopy (Leica TCS SP8).

### Glycan microarray

After treating the array slides with the Glycan Array Blocking Buffer (Creative Biochip Ltd.), the proteins (20, 10, 5 and 2.5 µg/mL) in the Glycan Array Assay Buffer (Creative Biochip Ltd.) were applied to the array slide surface. After 1 h incubation, the arrays were washed with the wash buffer (20 mmol/L Tris-HCl, 150 mM NaCl, 2 mM CaCl_2_, 2 mM MgCl_2_, 0.05% Tween-20, pH 7.4) twice for 5 min each time. The primary antibody (1:100 dilution) was applied to the array slide surface. After 1 h incubation, the arrays were washed with the wash buffer twice for 5 min each time. The secondary antibody was applied to the array slide surface. After 1 h incubation, the arrays were washed with the wash buffer and then deionized water. Finally, the slides were scanned with the microarray scanner LuxScan-10K/A at an excitation wavelength of 532 nm and evaluated by the Mapix microarray image analysis software. In this experiment, PC1 is the biotinylated polyethylene glycol used as a positive control for detecting eHA glycan binding, while PC3 is the mouse IgG used as a positive control for detecting tHA glycan binding. The negative control (NC) consisted of the sample buffer.

### CD experiments

The thermostabilities of the 1918 H1, H3N2 HA, InH5 HA, IBV HA (B/Austria/1359417/2021), tHA and eHA proteins were tested by CD spectroscopy. The spectra were obtained in PBS using a 1 cm optical path length cell. The protein concentration was 0.2 mg/mL. The temperature was increased by 0.5 °C/min. Thermal denaturation curves were determined by monitoring the CD value at 218 nm. The data were analyzed using Origin.8 software.

### Trypsin susceptibility assays

Purified HA0 in a buffer of pH 8.0 (20 mM Tris-HCl and 50 mM NaCl) was diluted to 1 mg/mL and 10 μL protein was added to each tube. The L-1-Tosylamido-2-phenylethyl chloromethyl ketone (TPCK)-treated trypsin (Sigma, concentration: 1 mg/mL) was added to each sample to a final concentration of 2.5 μg/mL and the digestion was performed at 37 °C. At time points of 0, 5, 10, 20, 40, 80 and 120 min, the digestion was stopped by adding a 5 × sodium dodecyl sulfate (SDS) loading buffer containing dithiothreitol (DTT) and boiled for 10 min. Samples were then loaded onto a 12% sodium dodecyl sulfate – polyacrylamide gel electrophoresis (SDS-PAGE) gel. For the low-pH digestion experiment, the HA0 was first digested with trypsin at 4 °C overnight and the extra trypsin was removed by gel filtration. The cleaved HA0 in PBS was diluted to 0.45 mg/mL and 40 μL protein was added to each tube. The 0.1 M citric acid was added to each sample and adjusted to a different pH, and the cleaved HA0 was incubated at 37 °C for 5 min. Then cleaved HA0 was incubated at pH 7.4 by using 0.5 M Tris-HCl to adjust the pH. The TPCK-treated trypsin (Sigma, concentration: 1 mg/mL) was added to each sample to a final concentration of 22.5 μg/mL and the digestion was performed at 20 °C for 1 h. The digestion was stopped by adding a 5 × SDS loading buffer containing DTT and boiled for 10 min. Samples were then loaded onto a 12% SDS-PAGE gel.

### Syncytia formation assay

The syncytia formation assay was performed in 12-well plates. The plasmids containing tHA were transfected into BHK-21 cells. The following day, the cells were washed 2–3 times with pre-warmed PBS at 37 °C, then washed once with pre-warmed TPCK-treated trypsin. Next, the cells were added with TPCK-treated trypsin and incubated at 37 °C for 15 min. and subsequently exposed to pH adjusted PBS ranging from pH 4.8 to 6.8 for 4 min. After aspiration of pH adjusted PBS, the cells were washed and incubated with dulbecco’s modified eagle medium (DMEM) supplemented with 10% fetal bovine serum (FBS) for 6 h at 37 °C. The cells were then fixed using 4% paraformaldehyde. Staining was performed using the DAPI for 1 h.

### Phylogenetic analysis and sequence alignment

Phylogenetic trees were constructed using the MEGA program (version 11) [56,57]. Maximum-likelihood analysis in combination with 1000 bootstrap replicates was used to derive trees. Evolutionary analyses were conducted in MEGA11. The following representative HA sequences were used to generate the phylogenetic tree of H1 to H18 and IBV HA subtypes: A/California/04/2009 (H1N1, GenBank accession No. ACP41105.1), A/Singapore/1/1957 (H2N2, GenBank accession No. ACF54477.1), A/Aichi/2/1968 (H3N2, GenBank accession No. AAA43239.1), A/duck/Czechoslovakia/1956 (H4N6, GenBank accession No. ACZ48536.1), A/duck/Malaysia/F119-3/97 (H5N3, GenBank accession No. AAG38534.1), A/chicken/California/431/2000 (H6N2, GenBank accession No. AAM69993.1), A/turkey/Italy/214845/2002 (H7N3, GenBank accession No. AAT37403.1), A/turkey/Ontario/6118/1968 (H8N4, GenBank accession No. BAF43468.1), A/swine/HongKong/9/98 (H9N2, GenBank accession No. AAL14080.1), A/chicken/Germany/N/1949 (H10N7, UniProtKB accession No. Q0A448.1), A/duck/England/1/1956 (H11N6, UniProtKB accession No. P04661.3), A/duck/Alberta/60/1976 (H12N5, GenBank accession No. ACZ48520.1), A/gull/Maryland/704/1977 (H13N6, UniProtKB accession No. P13103.3), A/mallard/Astrakhan/263/1982 (H14N5, GenBank accession No. ABI84453.1), A/shearwater/West Australia/2576/79 (H15N9, GenBank accession No. AAA96134.1), A/black-headed gull/Sweden/2/99 (H16N3, GenBank accession No. AAV91214.1), A/little yellow-shouldered bat/Guatemala/060/2010 (H17N10, GenBank accession No. AFC35438.1), A/flat-faced bat/Peru/033/2010 (H18N11, GenBank accession No. AGX84934.1), B/Victoria/2/1987 (B/Vic, UniProtKB accession No. P22092.1), and B/Yamagata/16/1988 (B/Yam, GenBank accession No. ABL77255.1).

## Supporting information

S1 Fig N-terminal amino acid sequences of the HA2 of tHA and eHA, in comparison with the fusion peptides among H1-H18 and IBV. The residues highlighted in red are completely conserved.
(TIF)

S2 Fig Glycan microarray of the recombinant proteins. Glycan microarray analyses of the tHA and eHA proteins. The PC1 was used as a positive control in the detection of eHA glycans binding. The PC3 was used as a positive control in the detection of tHA glycan binding. The negative control (NC) is the sample buffer used for HA function.
(TIF)

S3 Fig The hemagglutination assay of the recombinant proteins. Recombinant HA proteins starting at 10 μg serially diluted two-fold were applied in hemagglutination assay with 1% chicken or 2% guinea pig RBCs. The H3N2 HA and InH5 HA proteins were used as positive controls. PBS was used as negative control. All experiments were performed in triplicate, and one representative result is shown in S3 Fig.
(TIF)

S4 Fig Cryo-EM data processing of the tHA *apo.* (A) Representative cryo-EM micrograph and 2D classes. (B) The gold-standard Fourier shell correlation (FSC) curves of the final EM map and the viewing direction distribution plot for the tHA *apo* protein. (C) Workflow for the tHA *apo* protein 3D reconstructions.
(TIF)

S5 Fig Cryo-EM data processing of the eHA *apo.* (A) Representative cryo-EM micrograph and 2D classes. (B) The gold-standard FSC curves of the final EM map and the viewing direction distribution plot for the eHA *apo* protein. (C) Workflow for the eHA *apo* protein 3D reconstructions.
(TIF)

S6 Fig The overall structures of tHA and eHA are compared with that of IAV (A/Singapore/H2011.447/2011 PDB code:4WE8), IBV (B/Brisbane/60/2008 PDB code: 4FQM), respectively. (A) Trimer structure comparison of tHA (tHA is marked with different colors according to the domain.) and IAV HA (gray). RMSD value is 29.621 Å. Monomer structure comparison of tHA and IAV HA (gray). RMSD value is 5.144 Å. (B) Trimer structure comparison of tHA and IBV HA (gray). RMSD value is 29.890 Å. Monomer structure comparison of tHA and IBV HA (gray). RMSD value is 7.729 Å. (C) Trimer structure comparison of eHA (eHA is marked with different colors according to the domain.) and IAV HA (gray). RMSD value is 6.332 Å. Monomer structure comparison of eHA and IAV HA (gray). RMSD value is 5.409 Å. (D) Trimer structure comparison of eHA and IBV HA (gray). RMSD value is 3.256 Å. Monomer structure comparison of eHA and IBV HA (gray). RMSD value is 3.078 Å. Structure comparison shows that the structure of tHA and eHA are different from that of other HA structures.
(TIF)

S7 Fig Overview of the R subdomain structures in tHA, IAV (A/Singapore/H2011.447/2011 code:4WE8) and IBV (B/Brisbane/60/2008 PDB code: 4FQM). (A) Cartoon representation of the tHA R subdomain structure. (B) Cartoon representation of the eHA R subdomain structure. (C) Cartoon representation of the IAV R subdomain structure. (D) Cartoon representation of the IBV R subdomain structure. (E) Topology diagram of the tHA R subdomain following the same coloring scheme as in the cartoon representation. (F) Topology diagram of the eHA R subdomain following the same coloring scheme as in the cartoon representation. (G) Topology diagram of the IAV R subdomain following the same coloring scheme as in the cartoon representation. (H) Topology diagram of the IBV R subdomain following the same coloring scheme as in the cartoon representation.
(TIF)

S8 Fig Cryo-EM structural analysis of the α2–3 SA receptor with tHA protein complex. (A-B) Representative cryo-EM micrograph and 2D classes. (C-D) The gold-standard FSC curves of the final EM map and the viewing direction distribution plot for the α2–3 SA receptor with tHA protein complex. (E) Workflow for the α2–3 SA receptor with tHA protein complex 3D reconstructions.
(TIF)

S9 Fig Cryo-EM structural analysis of the α2–6 SA receptor with tHA protein complex. (A-B) Representative cryo-EM micrograph and 2D classes. (C-D) The gold-standard FSC curves of the final EM map and the viewing direction distribution plot for the α2–6 SA receptor with tHA protein complex. (E) Workflow for the α2–6 SA receptor with tHA protein complex 3D reconstructions.
(TIF)

S10 Fig 2Fo-Fc maps for the glycan receptors in the tHA/receptor complex structures. (A) tHA/avian receptor analog and (B) tHA/human receptor analog. The panels show portions of 2Fo-Fc electron density maps for these glycan receptor analogs contoured at 1.4 s and 1.0 s sigma, respectively, and the figures were drawn by Pymol software. The 2Fo-Fc maps were generated by FFT program in CCP4 software.
(TIF)

S11 Fig Cryo-EM structural analysis of the GM2 with eHA protein complex. (A-B) Representative cryo-EM micrograph and 2D classes. (C-D) The gold-standard FSC curves of the final EM map and the viewing direction distribution plot for the GM2 receptor with eHA protein complex. (E) Workflow for the GM2 receptor with eHA protein complex 3D reconstructions.
(TIF)

S12 Fig The surface electrostatic potential of sialic acid-binding grooves of tHA, eHA, IAV and IBV HAs. (TIF)

S1 Table Amino acid sequence identities among tHA, eHA, IAV HAs and IBV HAs. (DOCX)

S2 Table Structure similarity between tHA, eHA, IAV HAs and IBV HA. (DOCX)

S3 Table Hydrophobic interactions between IAV HA2 and adjacent IAV monomer molecule. (DOCX)

S4 Table Hydrophobic interactions between IBV HA2 and adjacent IBV monomer molecule. (DOCX)

S5 Table Statistical table of atomic contacts between receptors and tHA. (DOCX)

S6 Table Cryo-EM data collection, refinement and validation statistics. (DOCX)
