# Supplementary figures and images for: The hemagglutinin-like proteins of basal vertebrate influenza-like viruses exhibit sialic-acid receptor binding disparity and their structural bases
Source: PLoS Pathog. 2025 Nov 26;21(11):e1013640. doi: 10.1371/journal.ppat.1013640 (PMC12654924; doi:10.1371/journal.ppat.1013640)

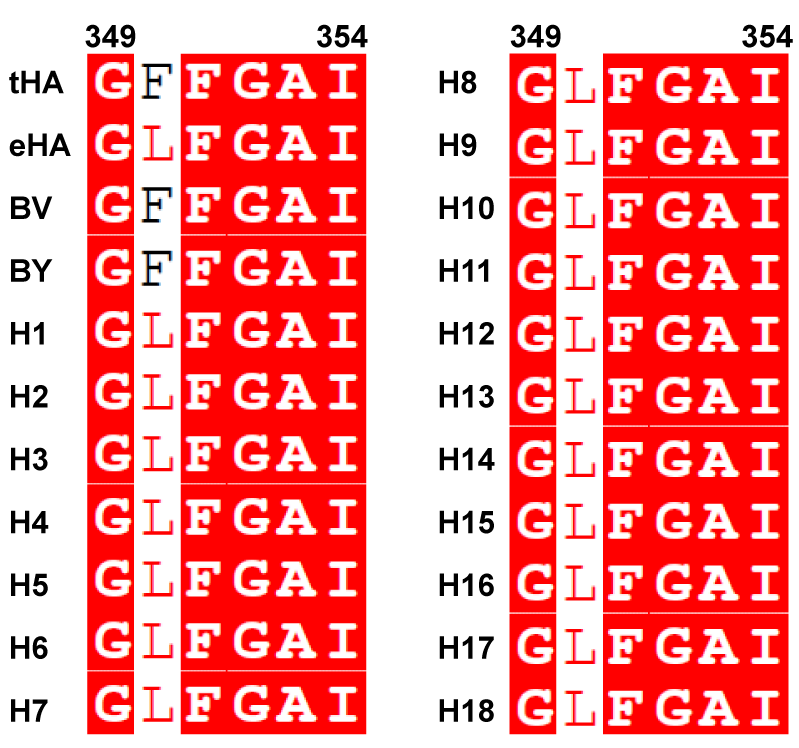

Supplement: S1 Fig — The residues highlighted in red are completely conserved. (TIF) [file ppat.1013640.s001.tif]

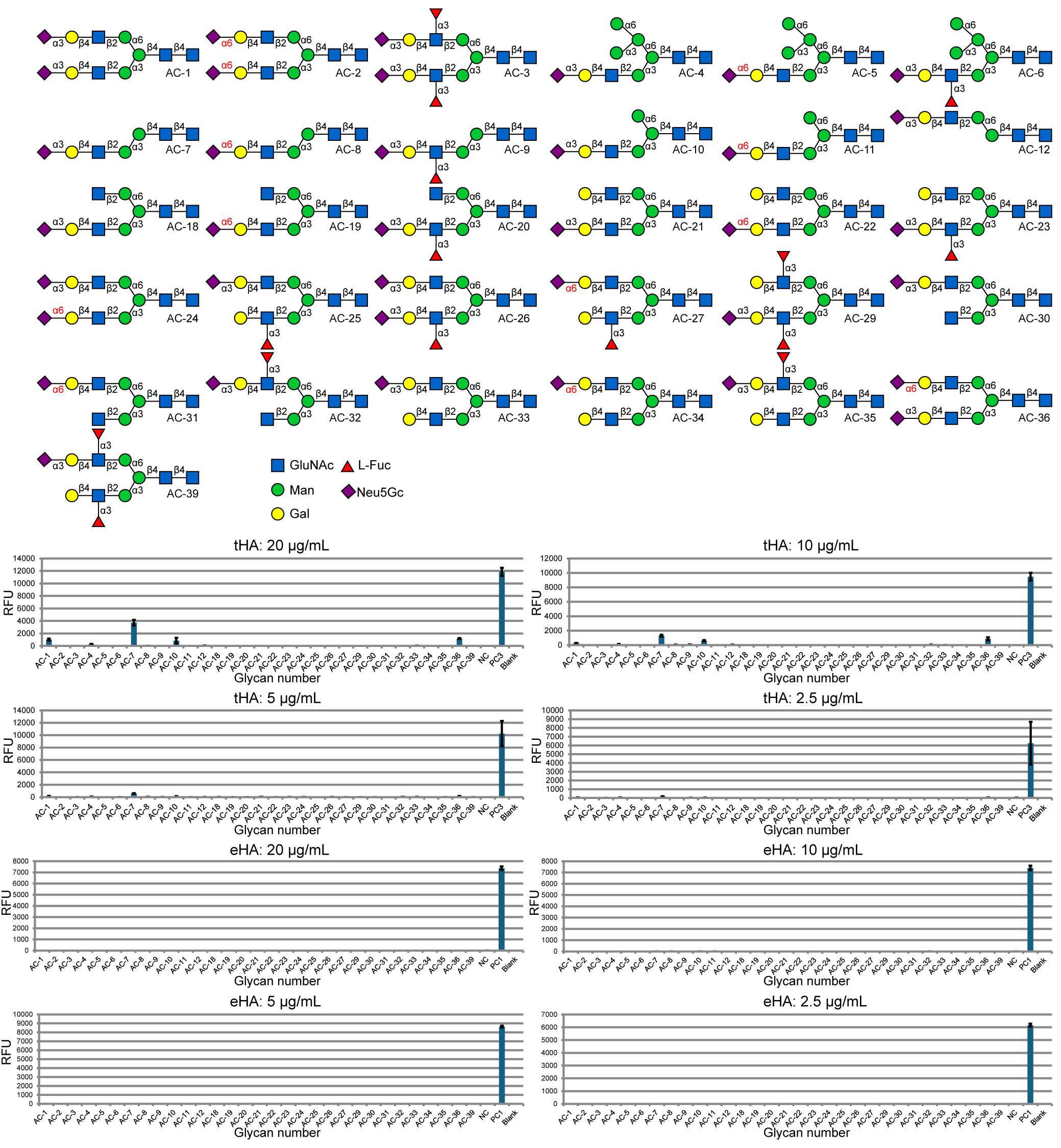

Supplement: S2 Fig — Glycan microarray analyses of the tHA and eHA proteins. The PC1 was used as a positive control in the detection of eHA glycans binding. The PC3 was used as a positive control in the detection of tHA glycan binding. The negative control (NC) is the sample buffer used for HA function. (TIF) [file ppat.1013640.s002.tif]

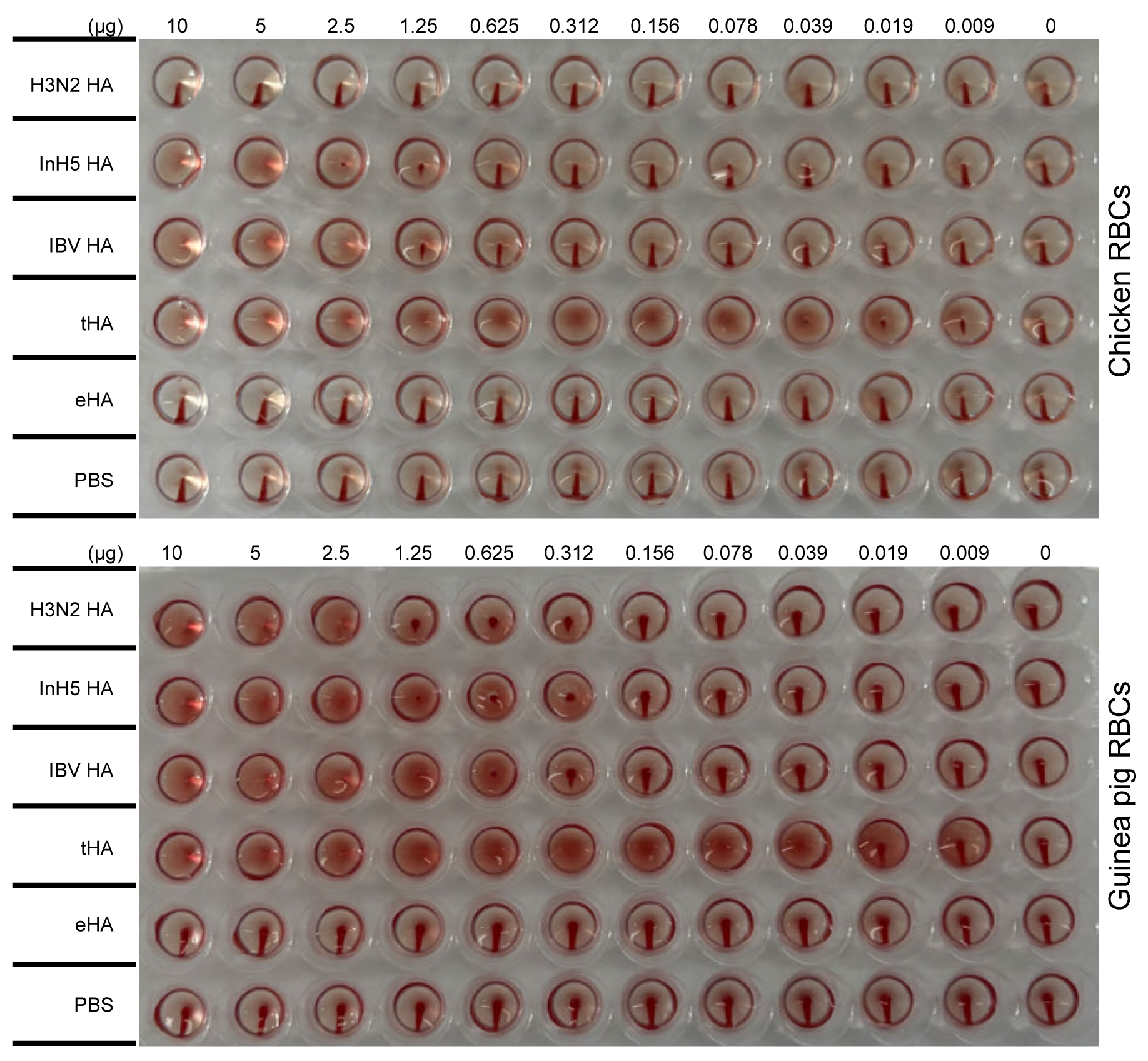

Supplement: S3 Fig — Recombinant HA proteins starting at 10 μg serially diluted two-fold were applied in hemagglutination assay with 1% chicken or 2% guinea pig RBCs. The H3N2 HA and InH5 HA proteins were used as positive controls. PBS was used as negative control. All experiments were performed in triplicate, and one representative result is shown in S3 Fig. (TIF) [file ppat.1013640.s003.tif]

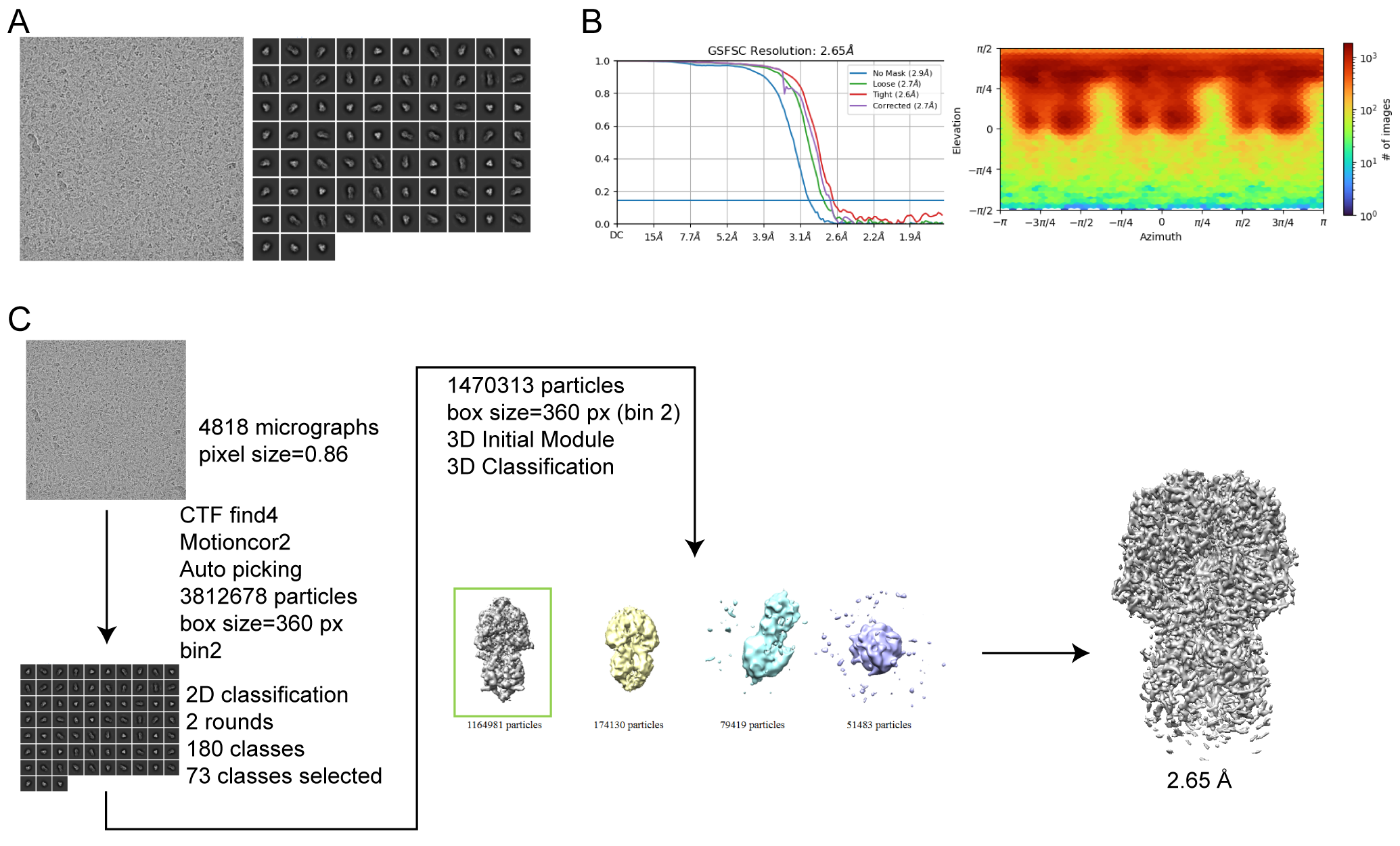

Supplement: S4 Fig — (A) Representative cryo-EM micrograph and 2D classes. (B) The gold-standard Fourier shell correlation (FSC) curves of the final EM map and the viewing direction distribution plot for the tHA apo protein. (C) Workflow for the tHA apo protein 3D reconstructions. (TIF) [file ppat.1013640.s004.tif]

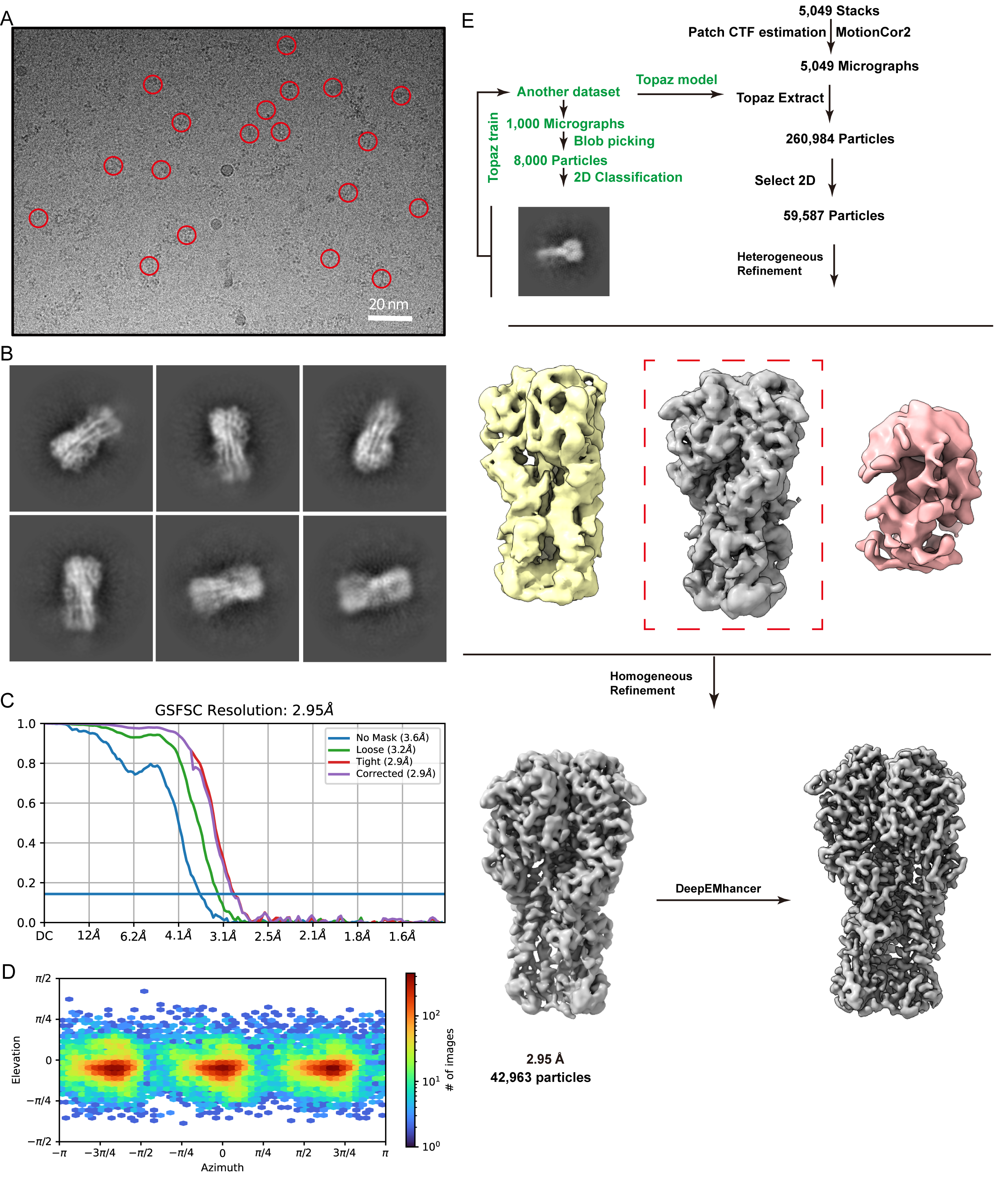

Supplement: S5 Fig — (A) Representative cryo-EM micrograph and 2D classes. (B) The gold-standard FSC curves of the final EM map and the viewing direction distribution plot for the eHA apo protein. (C) Workflow for the eHA apo protein 3D reconstructions. (TIF) [file ppat.1013640.s005.tif]

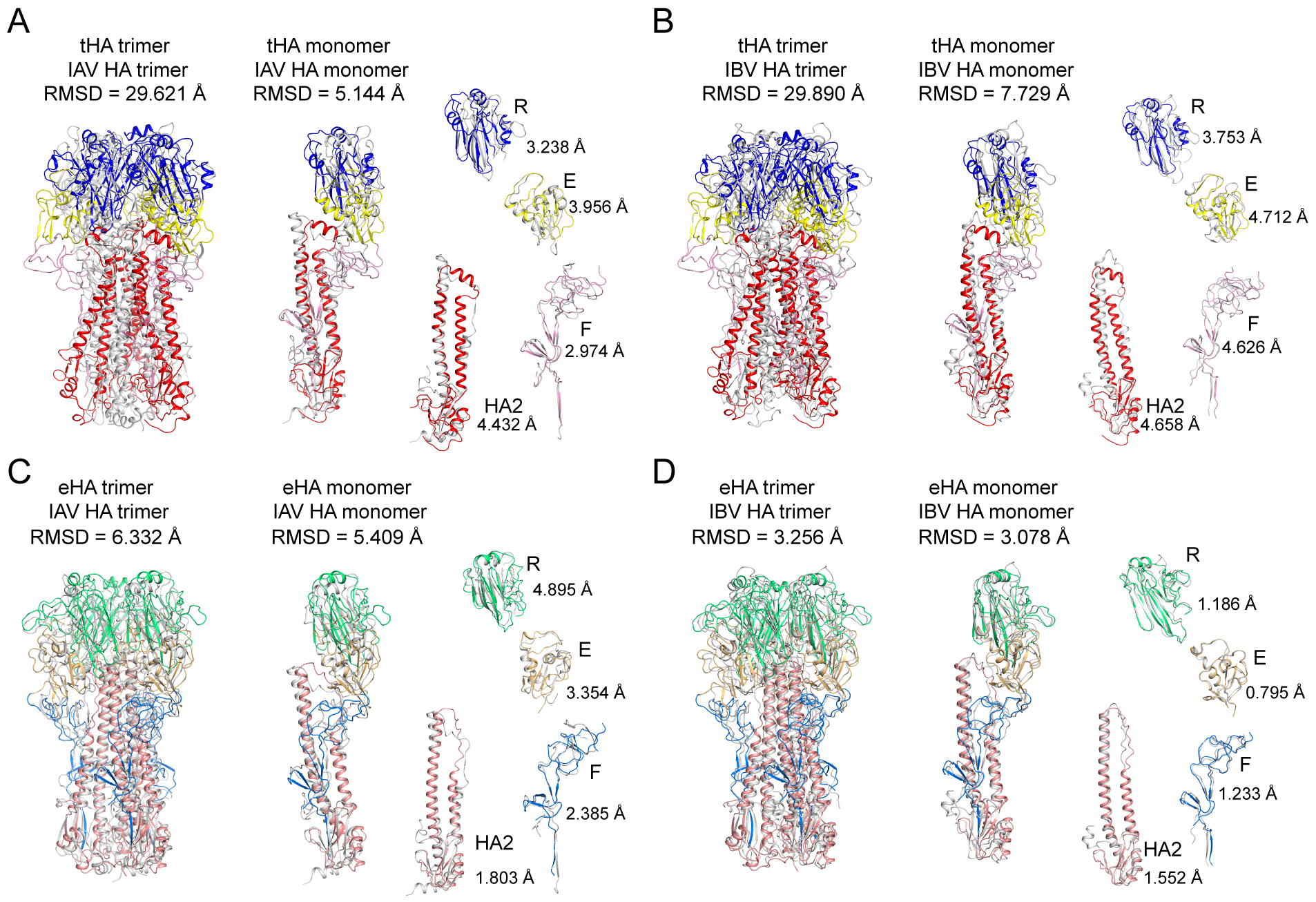

Supplement: S6 Fig — (A) Trimer structure comparison of tHA (tHA is marked with different colors according to the domain.) and IAV HA (gray). RMSD value is 29.621 Å. Monomer structure comparison of tHA and IAV HA (gray). RMSD value is 5.144 Å. (B) Trimer structure comparison of tHA and IBV HA (gray). RMSD value is 29.890 Å. Monomer structure comparison of tHA and IBV HA (gray). RMSD value is 7.729 Å. (C) Trimer structure comparison of eHA (eHA is marked with different colors according to the domain.) and IAV HA (gray). RMSD value is 6.332 Å. Monomer structure comparison of eHA and IAV HA (gray). RMSD value is 5.409 Å. (D) Trimer structure comparison of eHA and IBV HA (gray). RMSD value is 3.256 Å. Monomer structure comparison of eHA and IBV HA (gray). RMSD value is 3.078 Å. Structure comparison shows that the structure of tHA and eHA are different from that of other HA structures. (TIF) [file ppat.1013640.s006.tif]

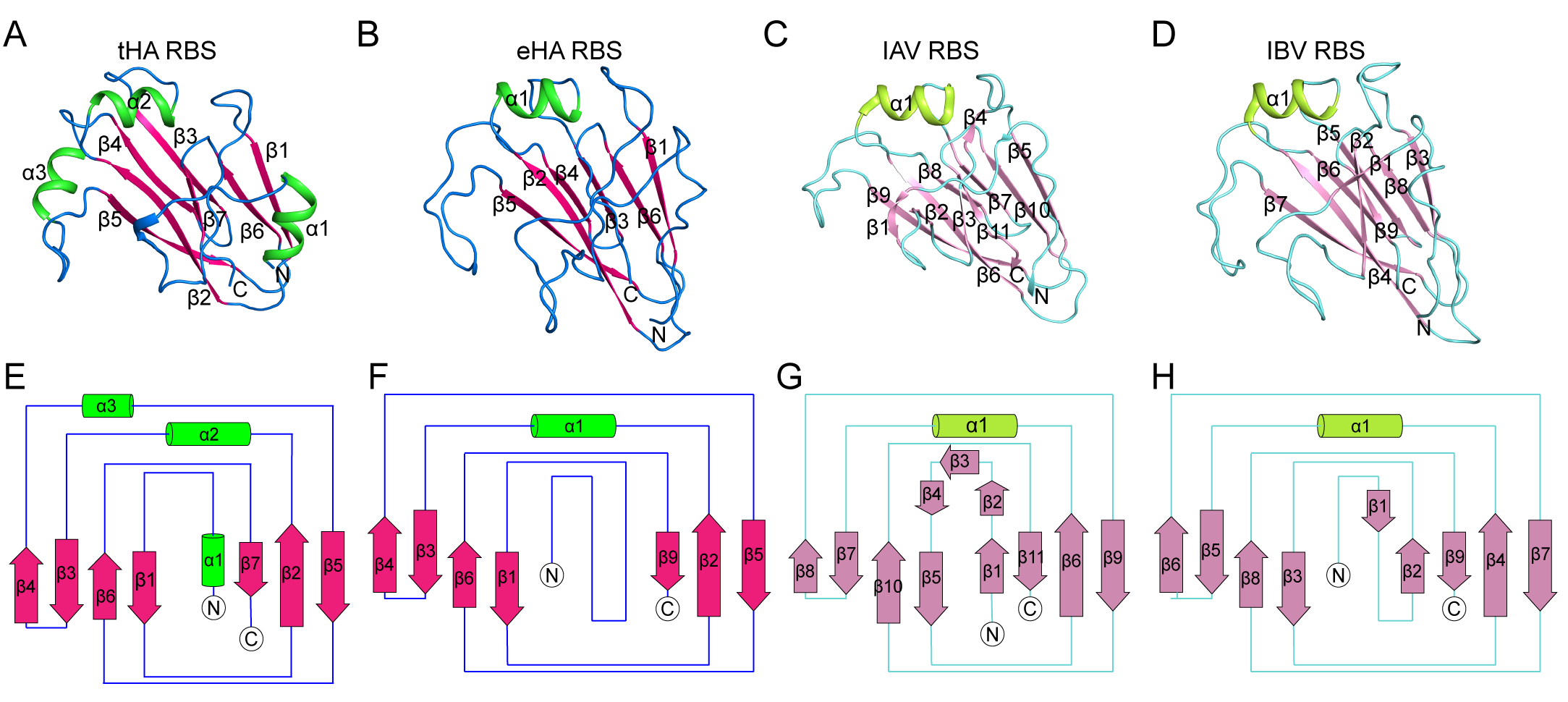

Supplement: S7 Fig — (A) Cartoon representation of the tHA R subdomain structure. (B) Cartoon representation of the eHA R subdomain structure. (C) Cartoon representation of the IAV R subdomain structure. (D) Cartoon representation of the IBV R subdomain structure. (E) Topology diagram of the tHA R subdomain following the same coloring scheme as in the cartoon representation. (F) Topology diagram of the eHA R subdomain following the same coloring scheme as in the cartoon representation. (G) Topology diagram of the IAV R subdomain following the same coloring scheme as in the cartoon representation. (H) Topology diagram of the IBV R subdomain following the same coloring scheme as in the cartoon representation. (TIF) [file ppat.1013640.s007.tif]

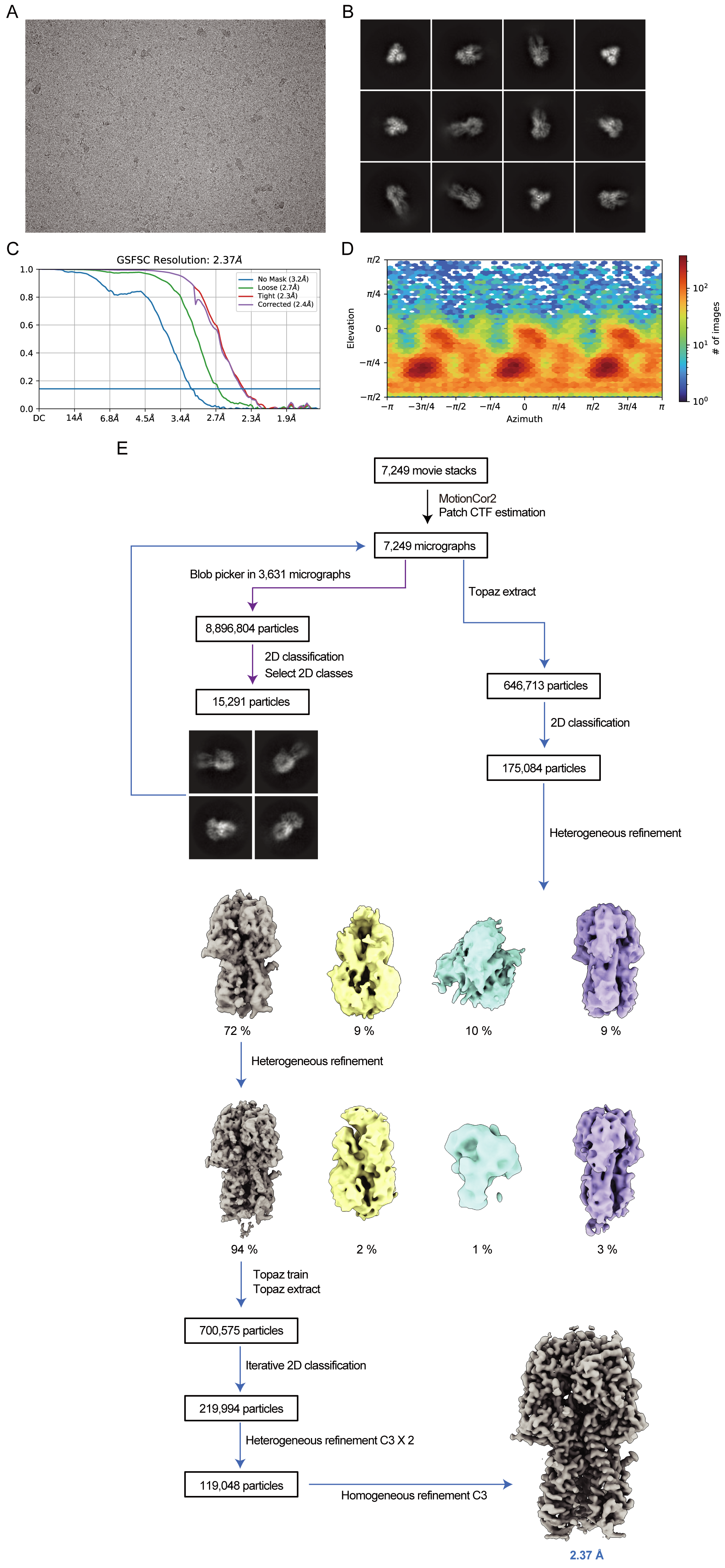

Supplement: S8 Fig — (A-B) Representative cryo-EM micrograph and 2D classes. (C-D) The gold-standard FSC curves of the final EM map and the viewing direction distribution plot for the α2–3 SA receptor with tHA protein complex. (E) Workflow for the α2–3 SA receptor with tHA protein complex 3D reconstructions. (TIF) [file ppat.1013640.s008.tif]

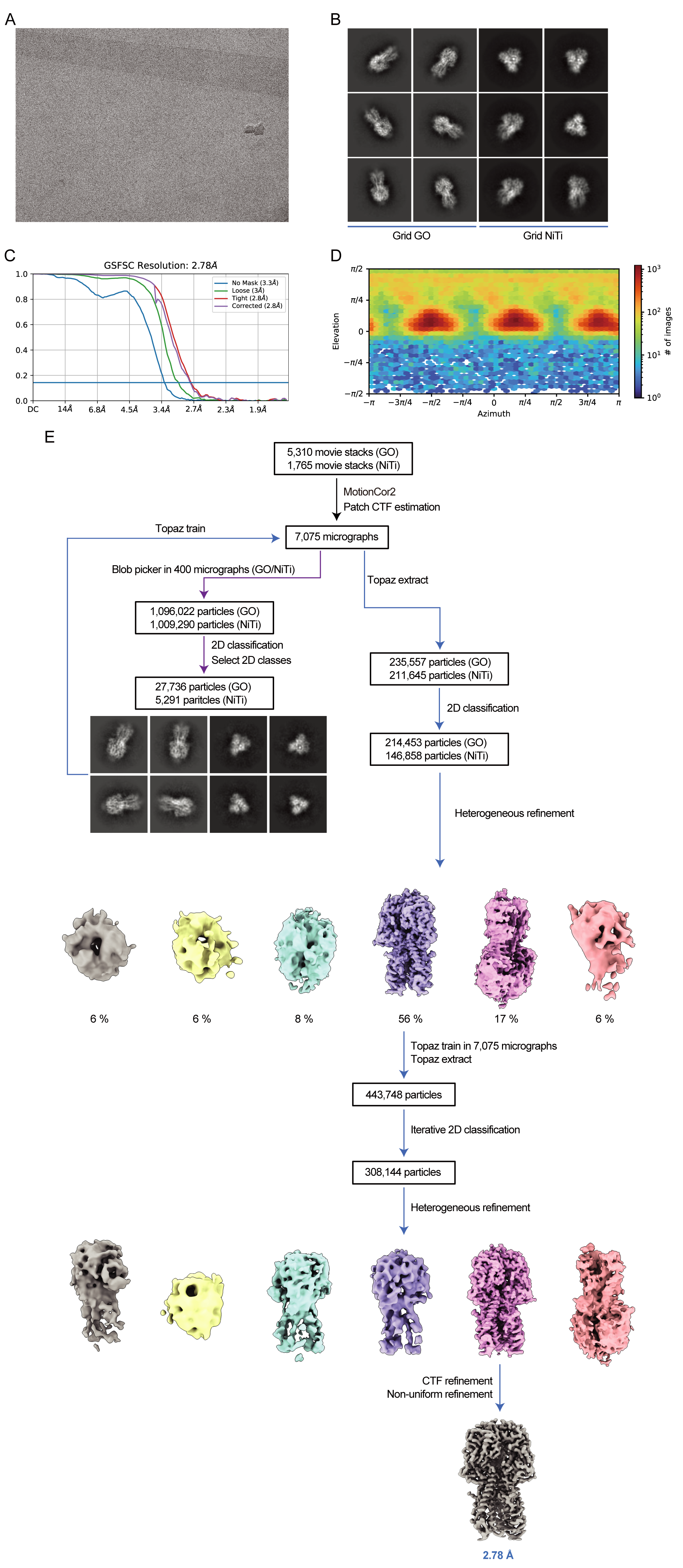

Supplement: S9 Fig — (A-B) Representative cryo-EM micrograph and 2D classes. (C-D) The gold-standard FSC curves of the final EM map and the viewing direction distribution plot for the α2–6 SA receptor with tHA protein complex. (E) Workflow for the α2–6 SA receptor with tHA protein complex 3D reconstructions. (TIF) [file ppat.1013640.s009.tif]

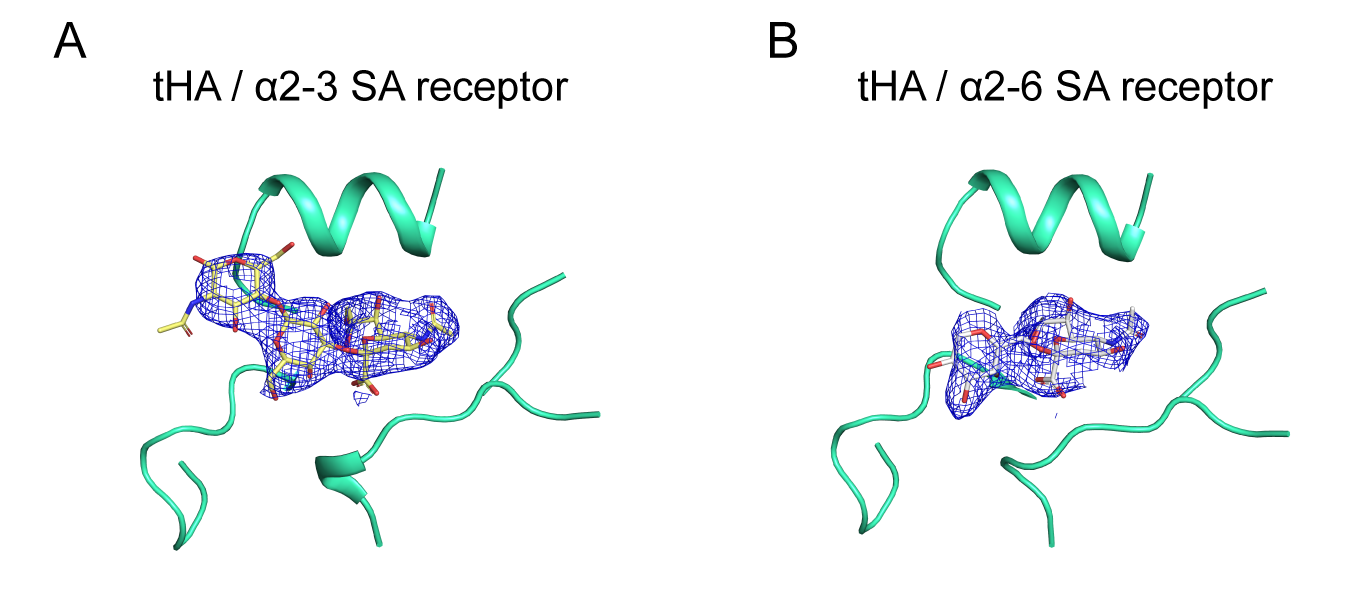

Supplement: S10 Fig — (A) tHA/avian receptor analog and (B) tHA/human receptor analog. The panels show portions of 2Fo-Fc electron density maps for these glycan receptor analogs contoured at 1.4 s and 1.0 s sigma, respectively, and the figures were drawn by Pymol software. The 2Fo-Fc maps were generated by FFT program in CCP4 software. (TIF) [file ppat.1013640.s010.tif]

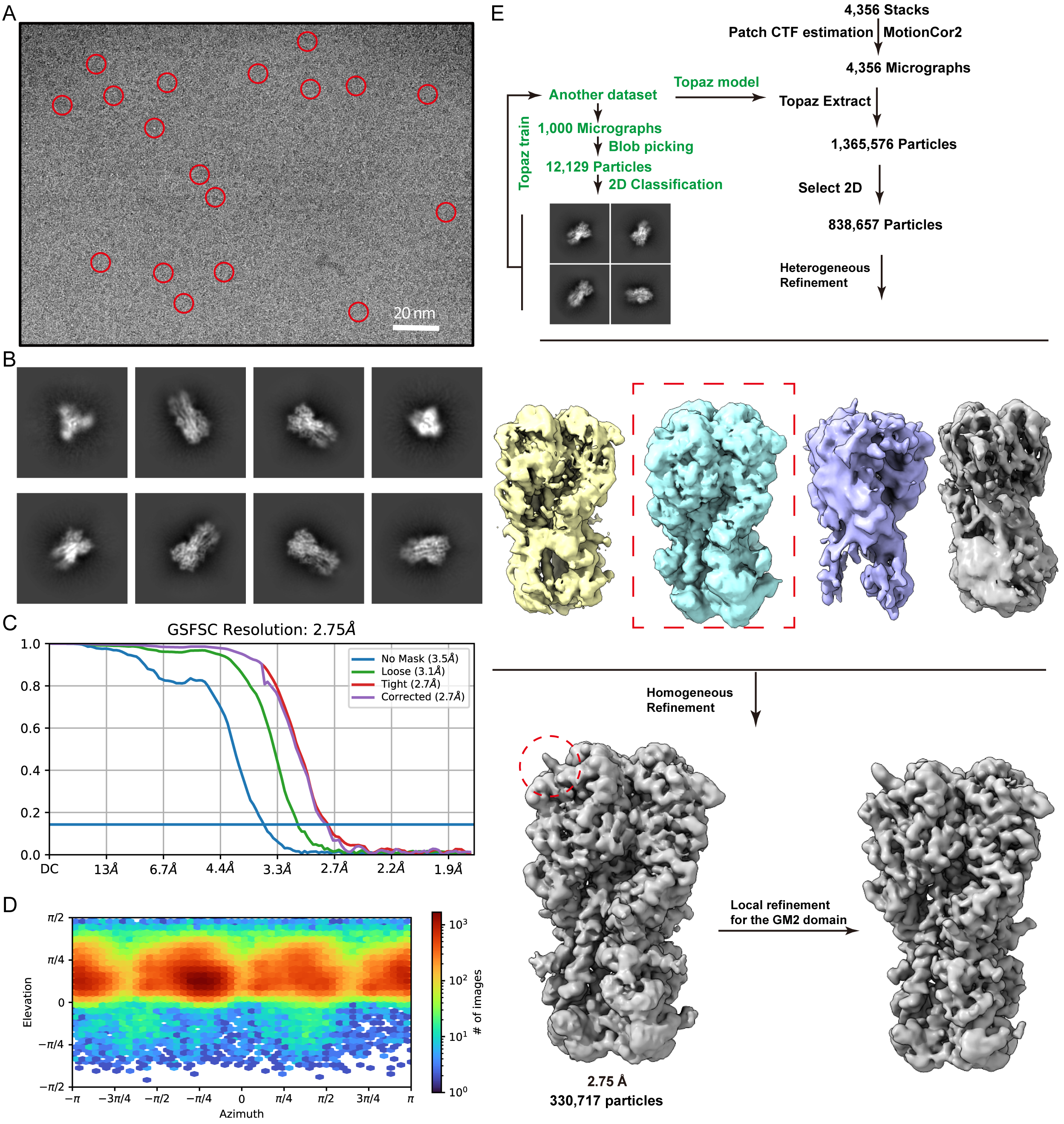

Supplement: S11 Fig — (A-B) Representative cryo-EM micrograph and 2D classes. (C-D) The gold-standard FSC curves of the final EM map and the viewing direction distribution plot for the GM2 receptor with eHA protein complex. (E) Workflow for the GM2 receptor with eHA protein complex 3D reconstructions. (TIF) [file ppat.1013640.s011.tif]

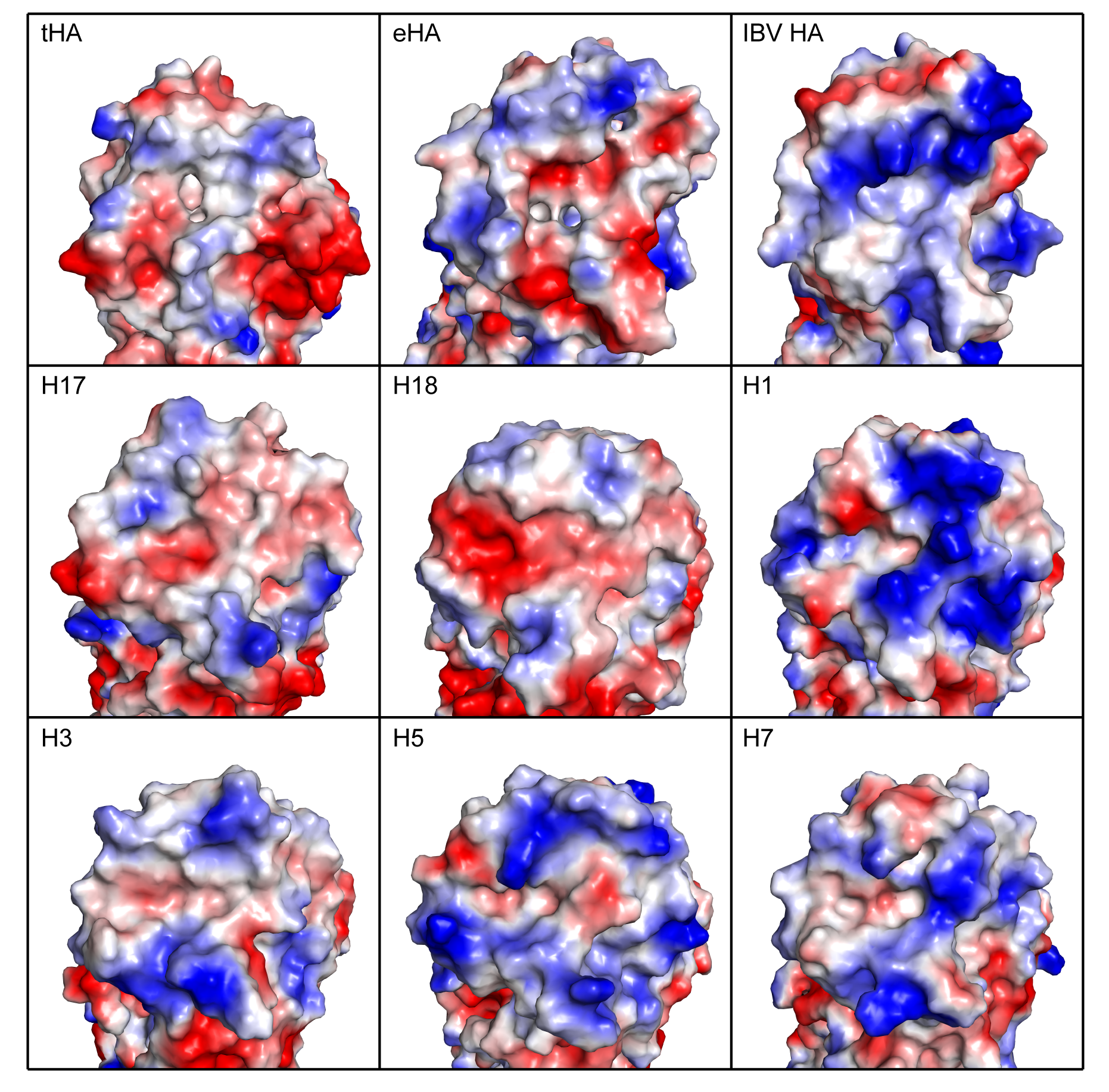

Supplement: S12 Fig — (TIF) [file ppat.1013640.s012.tif]
