# Supplementary material for: The hemagglutinin-like proteins of basal vertebrate influenza-like viruses exhibit sialic-acid receptor binding disparity and their structural bases
Source: PLoS Pathog. 2025 Nov 26;21(11):e1013640. doi: 10.1371/journal.ppat.1013640 (PMC12654924; doi:10.1371/journal.ppat.1013640)
Supplement: S1 Table — (DOCX) [file ppat.1013640.s013.docx]

| Identity | tHA | eHA | H1 | H2 | H3 | H4 | H5 | H6 | H7 | H8 | H9 | H10 | H11 | H12 | H13 | H14 | H15 | H16 | H17 | H18 | B/Vic |
| --- | --- | --- | --- | --- | --- | --- | --- | --- | --- | --- | --- | --- | --- | --- | --- | --- | --- | --- | --- | --- | --- |
| tHA |  |  |  |  |  |  |  |  |  |  |  |  |  |  |  |  |  |  |  |  |  |
| eHA | 20.97 |  |  |  |  |  |  |  |  |  |  |  |  |  |  |  |  |  |  |  |  |
| H1 | 22.57 | 25.13 |  |  |  |  |  |  |  |  |  |  |  |  |  |  |  |  |  |  |  |
| H2 | 22.15 | 25.94 | 63.78 |  |  |  |  |  |  |  |  |  |  |  |  |  |  |  |  |  |  |
| H3 | 24.27 | 23.09 | 43.13 | 40.84 |  |  |  |  |  |  |  |  |  |  |  |  |  |  |  |  |  |
| H4 | 24.52 | 23.17 | 42.66 | 39.82 | 65.49 |  |  |  |  |  |  |  |  |  |  |  |  |  |  |  |  |
| H5 | 21.88 | 26.54 | 65.37 | 76.24 | 42.68 | 42.03 |  |  |  |  |  |  |  |  |  |  |  |  |  |  |  |
| H6 | 23.54 | 25.51 | 58.10 | 58.83 | 40.28 | 41.96 | 62.19 |  |  |  |  |  |  |  |  |  |  |  |  |  |  |
| H7 | 22.96 | 23.60 | 40.18 | 39.79 | 48.07 | 49.47 | 42.71 | 41.05 |  |  |  |  |  |  |  |  |  |  |  |  |  |
| H8 | 22.34 | 26.92 | 48.51 | 48.32 | 41.57 | 43.08 | 51.76 | 51.06 | 39.93 |  |  |  |  |  |  |  |  |  |  |  |  |
| H9 | 19.90 | 26.75 | 48.68 | 49.73 | 37.52 | 40.00 | 49.47 | 51.76 | 38.87 | 58.91 |  |  |  |  |  |  |  |  |  |  |  |
| H10 | 22.47 | 25.51 | 42.88 | 42.96 | 49.91 | 49.91 | 43.84 | 41.05 | 64.41 | 41.65 | 39.86 |  |  |  |  |  |  |  |  |  |  |
| H11 | 24.01 | 25.51 | 53.44 | 53.81 | 42.91 | 41.71 | 57.35 | 54.67 | 40.56 | 50.18 | 50.62 | 42.36 |  |  |  |  |  |  |  |  |  |
| H12 | 20.28 | 24.57 | 46.38 | 47.43 | 42.29 | 42.46 | 48.76 | 50.70 | 41.65 | 66.25 | 60.53 | 41.73 | 49.82 |  |  |  |  |  |  |  |  |
| H13 | 20.86 | 26.11 | 49.74 | 46.29 | 39.69 | 39.62 | 50.18 | 47.89 | 38.42 | 48.06 | 50.18 | 39.02 | 58.27 | 49.29 |  |  |  |  |  |  |  |
| H14 | 23.80 | 24.27 | 41.32 | 39.37 | 64.79 | 75.35 | 42.43 | 43.06 | 48.25 | 41.32 | 38.68 | 48.78 | 42.21 | 40.77 | 41.00 |  |  |  |  |  |  |
| H15 | 23.67 | 23.21 | 39.66 | 40.55 | 47.84 | 48.70 | 41.87 | 41.18 | 78.77 | 38.86 | 37.17 | 64.16 | 39.90 | 42.66 | 39.10 | 49.91 |  |  |  |  |  |
| H16 | 22.09 | 25.55 | 49.65 | 45.76 | 38.58 | 39.58 | 48.85 | 46.75 | 36.97 | 47.36 | 49.03 | 37.68 | 57.50 | 49.12 | 78.80 | 39.90 | 35.70 |  |  |  |  |
| H17 | 22.70 | 25.13 | 49.82 | 52.83 | 37.98 | 36.95 | 51.50 | 49.47 | 37.02 | 46.83 | 44.70 | 34.56 | 46.48 | 45.68 | 46.40 | 38.93 | 35.30 | 47.09 |  |  |  |
| H18 | 22.70 | 26.80 | 49.47 | 51.42 | 37.39 | 37.94 | 53.53 | 50.44 | 38.18 | 46.38 | 43.56 | 36.20 | 45.87 | 44.88 | 46.70 | 37.67 | 38.00 | 46.83 | 61.06 |  |  |
| B/Vic | 22.54 | 42.83 | 28.40 | 26.35 | 25.25 | 25.55 | 28.33 | 26.98 | 26.60 | 27.32 | 28.02 | 25.72 | 26.86 | 27.95 | 26.90 | 25.08 | 25.40 | 27.44 | 25.55 | 27.41 |  |
| B/Yam | 23.19 | 42.81 | 28.55 | 26.10 | 25.34 | 26.32 | 28.76 | 27.24 | 26.40 | 27.87 | 28.50 | 25.64 | 27.92 | 27.24 | 27.20 | 25.38 | 25.60 | 28.64 | 26.52 | 27.16 | 95.56 |
