# Supplementary material for: The hemagglutinin-like proteins of basal vertebrate influenza-like viruses exhibit sialic-acid receptor binding disparity and their structural bases
Source: PLoS Pathog. 2025 Nov 26;21(11):e1013640. doi: 10.1371/journal.ppat.1013640 (PMC12654924; doi:10.1371/journal.ppat.1013640)
Supplement: S2 Table — (DOCX) [file ppat.1013640.s014.docx]

| HA | PDB ID | RMSD (Å) of the Cα atoms of one HA monomer | | | | | | | | | | | | | | | | | | | | | |
| --- | --- | --- | --- | --- | --- | --- | --- | --- | --- | --- | --- | --- | --- | --- | --- | --- | --- | --- | --- | --- | --- | --- | --- |
|  |  | tHA | eHA | H1 | H2 | H3 | H4 | H5 | H6 | H7 | H8 | H9 | H10 | H11 | H12 | H13 | H14 | H15 | H16 | H17 | H18 | B/Vic | B/Yam |
| tHA |  |  | 9.734 | 12.720 | 7.240 | **5.144** | 6.002 | 6.496 | 8.024 | 5.542 | 7.979 | 8.816 | 5.879 | 6.674 | 6.555 | 5.668 | 6.125 | 7.195 | 10.649 | 6.774 | 11.535 | **7.729** | 7.197 |
| eHA |  |  |  | 6.635 | 7.776 | **5.409** | 5.478 | 6.685 | 7.210 | 5.544 | 6.167 | 5.800 | 5.677 | 5.206 | 6.128 | 5.613 | 5.593 | 5.533 | 5.392 | 6.155 | 6.455 | **3.078** | 3.021 |
| H1 | 3AL4 |  |  |  | 0.669 | **2.365** | 2.314 | 0.848 | 1.101 | 2.701 | 1.181 | 1.448 | 2.413 | 0.968 | 1.668 | 1.717 | 2.128 | 2.530 | 1.867 | 1.326 | 0.966 | **6.293** | 6.221 |
| H2 | 2WRC |  |  |  |  | **2.410** | 2.360 | 0.582 | 0.932 | 2.682 | 1.146 | 1.472 | 2.483 | 1.014 | 1.589 | 1.769 | 2.172 | 2.586 | 1.855 | 1.454 | 1.057 | **6.335** | 6.619 |
| H3 | **4WE8** |  |  |  |  |  | **0.476** | **2.349** | **2.582** | **1.138** | **2.454** | **2.175** | **1.216** | **2.026** | **2.604** | **2.071** | **0.565** | **1.337** | **2.050** | **2.400** | **2.194** | **4.942** | **4.716** |
| H4 | 5XL3 |  |  |  |  |  |  | 2.284 | 2.529 | 1.111 | 2.407 | 2.163 | 1.336 | 2.015 | 2.581 | 2.083 | 0.436 | 1.459 | 2.024 | 2.349 | 2.035 | **4.992** | 4.637 |
| H5 | 1JSM |  |  |  |  |  |  |  | 0.817 | 2.737 | 1.436 | 1.141 | 2.632 | 0.912 | 1.480 | 2.135 | 2.095 | 2.786 | 2.240 | 1.967 | 1.524 | **6.255** | 6.198 |
| H6 | 4XKD |  |  |  |  |  |  |  |  | 2.774 | 1.323 | 1.253 | 2.729 | 0.943 | 1.483 | 2.163 | 2.205 | 3.066 | 2.212 | 1.859 | 1.577 | **6.045** | 5.949 |
| H7 | 1TI8 |  |  |  |  |  |  |  |  |  | 2.702 | 2.572 | 0.923 | 2.396 | 2.912 | 2.133 | 1.212 | 1.015 | 2.173 | 2.411 | 2.378 | **5.006** | 4.739 |
| H8 | 6V46 |  |  |  |  |  |  |  |  |  |  | 1.218 | 2.689 | 1.117 | 1.343 | 1.475 | 2.076 | 2.725 | 1.630 | 1.261 | 1.187 | **5.968** | 5.633 |
| H9 | 1JSD |  |  |  |  |  |  |  |  |  |  |  | 2.263 | 1.300 | 1.066 | 1.897 | 2.000 | 2.717 | 1.966 | 1.417 | 1.690 | **5.781** | 5.500 |
| H10 | 4CYV |  |  |  |  |  |  |  |  |  |  |  |  | 2.325 | 2.536 | 2.499 | 1.380 | 1.327 | 2.365 | 2.356 | 2.399 | **4.502** | 4.783 |
| H11 | 6V47 |  |  |  |  |  |  |  |  |  |  |  |  |  | 1.469 | 1.531 | 1.782 | 2.316 | 1.773 | 1.728 | 1.288 | **5.177** | 5.077 |
| H12 | 7A9D |  |  |  |  |  |  |  |  |  |  |  |  |  |  | 2.253 | 2.410 | 3.060 | 2.459 | 1.731 | 1.925 | **5.788** | 5.544 |
| H13 | 4KPQ |  |  |  |  |  |  |  |  |  |  |  |  |  |  |  | 1.850 | 2.159 | 0.442 | 1.385 | 1.518 | **5.541** | 5.324 |
| H14 | 3EYJ |  |  |  |  |  |  |  |  |  |  |  |  |  |  |  |  | 1.427 | 1.849 | 2.098 | 1.985 | **4.939** | 4.796 |
| H15 | 5TG8 |  |  |  |  |  |  |  |  |  |  |  |  |  |  |  |  |  | 2.294 | 2.604 | 2.249 | **5.303** | 5.287 |
| H16 | 4F23 |  |  |  |  |  |  |  |  |  |  |  |  |  |  |  |  |  |  | 1.413 | 1.547 | **5.661** | 5.243 |
| H17 | 4H32 |  |  |  |  |  |  |  |  |  |  |  |  |  |  |  |  |  |  |  | 1.419 | **4.656** | 4.428 |
| H18 | 4K3X |  |  |  |  |  |  |  |  |  |  |  |  |  |  |  |  |  |  |  |  | **5.180** | 5.517 |
| B/Vic | **4FQM** |  |  |  |  |  |  |  |  |  |  |  |  |  |  |  |  |  |  |  |  |  | **0.903** |
| B/Yam | 3BT6 |  |  |  |  |  |  |  |  |  |  |  |  |  |  |  |  |  |  |  |  |  | **–** |
