# Supplementary material for: The hemagglutinin-like proteins of basal vertebrate influenza-like viruses exhibit sialic-acid receptor binding disparity and their structural bases
Source: PLoS Pathog. 2025 Nov 26;21(11):e1013640. doi: 10.1371/journal.ppat.1013640 (PMC12654924; doi:10.1371/journal.ppat.1013640)
Supplement: S3 Table — (DOCX) [file ppat.1013640.s015.docx]

| IAV HA2 | IAV HA |
| --- | --- |
| 221P | 244L (6) 242 (3) |
| 331I | 332F (12) |
| 332F | 332F (8) |
| 402V | 111L (4), 236I (3), 260I (1) |
| 406I | 406I (3) |
| 409L | 395I (5), 413V (2), 409L (3) |
| 413V | 413V (3) |
| 420L | 421W (15), 420L (3) |
| 431L | 431L (1) |
| 500F | 496L (5), 500F (7) |
| Total | 84 |

The numbers in parentheses for IAV HA represent the number of hydrophobic interactions between the IAV HA2 residues with the adjacent IAV HA monomer molecule. Hydrophobic interaction was analyzed at a cutoff of 4.5 Å.
