# Supplementary material for: The hemagglutinin-like proteins of basal vertebrate influenza-like viruses exhibit sialic-acid receptor binding disparity and their structural bases
Source: PLoS Pathog. 2025 Nov 26;21(11):e1013640. doi: 10.1371/journal.ppat.1013640 (PMC12654924; doi:10.1371/journal.ppat.1013640)
Supplement: S4 Table — (DOCX) [file ppat.1013640.s016.docx]

| IBV HA2 | IBV HA |
| --- | --- |
| 350F | 463L (11), 464A (17) |
| 353I | 464A (6), 465L (5) |
| 354A | 464A (8) |
| 424I | 424I (3) |
| 427L | 413L (6), 424I (3), 427L (4), 431V (1) |
| 431V | 431V (2) |
| 434L | 410L (3) |
| 445L | 446A (2) |
| 449L | 449L (6) |
| 478V | 475P (1), 514F (3) |
| 480I | 514F (3) |
| Total | 84 |

The numbers in parentheses for IBV HA represent the number of hydrophobic interactions between the IBV HA2 residues with the adjacent IBV HA monomer molecule. Hydrophobic interaction was analyzed at a cutoff of 4.5 Å.
