# Supplementary material for: The hemagglutinin-like proteins of basal vertebrate influenza-like viruses exhibit sialic-acid receptor binding disparity and their structural bases
Source: PLoS Pathog. 2025 Nov 26;21(11):e1013640. doi: 10.1371/journal.ppat.1013640 (PMC12654924; doi:10.1371/journal.ppat.1013640)
Supplement: S5 Table — (DOCX) [file ppat.1013640.s017.docx]

| tHA | α2-3 glycans | α2-6 glycans |
| --- | --- | --- |
| G148 | Sia1 (1) | **–** |
| A149 | Sia1 (9,**1**) | Sia1 (6,**1**) |
| T150 | Sia1 (12,**1**) | Sia1 (10,**1**) |
| S151 | Sia1 (4,**2**), Gal2 (2) | Sia1 (7,**1**), Gal2 (3) |
| E152 | Gal2 (4,**1**) | Gal2 (2) |
| W161 | Sia1 (7) | Sia1 (8) |
| V163 | Sia1 (3) | Sia1 (3) |
| S190 | Sia1 (5) | Sia1 (5) |
| G195 | Sia1 (3) | **–** |
| V199 | Sia1 (6) | Sia1 (3) |
| N247 | Sia1 (5,**2**) | Sia1 (5,**2**) |
| Total | 61, 7 | 52, 5 |

“–” represents the residue in tHA does not contact with the corresponding residue in the α2-6 glycans. The numbers without underline in parentheses indicate the number of van der Waals force contacts between the pairs of residues. Underlined numbers with bold format suggest numbers of potential H-bonds between the pairs of residues. Van der Waals contact was analyzed at a cutoff of 4.5 Å and H-bonds at a cutoff of 3.5 Å.
