# Supplementary material for: The hemagglutinin-like proteins of basal vertebrate influenza-like viruses exhibit sialic-acid receptor binding disparity and their structural bases
Source: PLoS Pathog. 2025 Nov 26;21(11):e1013640. doi: 10.1371/journal.ppat.1013640 (PMC12654924; doi:10.1371/journal.ppat.1013640)
Supplement: S6 Table — (DOCX) [file ppat.1013640.s018.docx]

|  | tHA | tHA/α2-3 SA receptor | tHA/α2-6 SA receptor | eHA | eHA/GM2 |
| --- | --- | --- | --- | --- | --- |
| **Data Collection & Processing** |  |  |  |  |  |
| Magnification | 96K | 105K | 105K | 105k | 85K |
| Voltage (kV) | 300 | 300 | 300 | 300 | 300 |
| Electron exposure (e^–^/Å^2^) | 49 | 50 | 50 | 60 | 50 |
| Defocus range (μm) | -1.0~-2.0 | -1.0~-2.0 | -1.0~-2.0 | -1.0~-2.0 | -1.0~-2.0 |
| Pixel size (Å) | 0.86 | 0.85 | 0.85 | 0.69 | 0.89 |
| Symmetry imposed | C3 | C3 | C1 | C3 | C1 |
| Final particles images | 1,160,779 | 119,048 | 206,720 | 42,963 | 330,717 |
| Map resolution (Å) | 2.65 | 2.37 | 2.78 | 2.95 | 2.75 |
| FSC threshold | 0.143 | 0.143 | 0.143 | 0.143 | 0.143 |
| **Refinement** |  |  |  |  |  |
| Initial model used (PDB code) | 8WH7 | 9W44 | 8WIS | 9KED |  |
| EMDB | EMD-37532 | EMD-65618 | EMD-37567 | EMD-62290 | EMD-65498 |
| Non-hydrogen atoms | 11,367 | 11,459 | 11,429 | 11,277 |  |
| Protein residues | 1425 | 1425 | 1425 | 1473 |  |
| Validation |  |  |  |  |  |
| Clash score | 2.73 | 4.44 | 4.90 | 10.38 |  |
| Poor rotamers (%) | 0.00 | 3.89 | 0.00 | 4.45 |  |
| R.m.s. deviations |  |  |  |  |  |
| Bond lengths (Å) | 0.003 | 0.003 | 0.003 | 0.003 |  |
| Bond angles (°) | 0.498 | 0.495 | 0.486 | 0.690 |  |
| Ramachandran statistics (%) |  |  |  |  |  |
| Most favored | 96.77 | 98.32 | 97.40 | 92.95 |  |
| Allowed | 3.23 | 1.68 | 2.60 | 7.05 |  |
